# IFC-HFlowVAE: A self-enhancing generative framework with structural anchoring for imbalanced clinical data augmentation

**DOI:** 10.1371/journal.pone.0357260

**Published:** 2026-09-03

**Authors:** Lu Yuwen, Shuyu Chen

**Affiliations:** School of Big Data & Software Engineering, Chongqing University, Chongqing, China; Commonwealth Scientific and Industrial Research Organisation, AUSTRALIA

## Abstract

Class imbalance and limited minority-class samples remain major challenges for developing reliable clinical diagnostic models, as insufficient observations often fail to capture complex minority-class distributions. Existing augmentation methods either rely on heuristic interpolation or suffer from instability when learning sparse and heterogeneous medical tabular data. This study proposes IFC-HFlowVAE, an iterative feedback and consensus framework built upon a flow-enhanced heterogeneous variational autoencoder for minority-class data generation. The proposed framework first models mixed-type clinical attributes through HFlowVAE and then introduces a self-circulating refinement strategy to progressively improve generated samples. To alleviate degradation during iterative refinement, SMOTE-generated samples are incorporated as structural references, followed by a localized GMM-based refinement procedure that guides samples toward more representative minority-class regions. Comprehensive experiments on seven clinical tabular datasets demonstrate that IFC-HFlowVAE improves downstream classification performance compared with existing augmentation approaches. Furthermore, fidelity analyses show that the proposed framework better preserves challenging minority-class characteristics, particularly skewed and multimodal numerical distributions.

## Introduction

The rapid digitization of healthcare has created new opportunities for automated diagnosis and prognostic modeling. In high-stakes scenarios such as diabetes management, where prevalence is projected to reach 9.7% by 2030 [1], reliable screening systems are essential for improving clinical efficiency. Recent advances in medical AI have demonstrated strong capabilities in extracting meaningful representations from complex medical data, particularly in image-based tasks such as segmentation and classification [2–4]. However, these approaches are not directly applicable to heterogeneous tabular data, where class imbalance and diverse attribute types remain major challenges [5]. Although imbalance is traditionally regarded as a sample scarcity problem [6], recent studies indicate that small and imbalanced clinical datasets impose fundamental difficulties on standard machine learning models [7]. We refer to this limitation as structural overfitting, where insufficient minority representation prevents models from learning discriminative pathological patterns and causes the minority manifold to be biased toward the majority distribution [8]. Such degradation is particularly severe when class overlap exists in clinical tabular data [9–12].

Traditional augmentation approaches, such as SMOTE and its variants [13–15], alleviate imbalance through interpolation-based sample generation. However, these heuristic strategies may introduce unrealistic synthetic patterns and increase sample density in overlapping regions [16,17]. Deep Generative Models (DGMs), including VAEs [18,19] and GAN-based models [20,21], provide a promising alternative by learning nonlinear data distributions. VAEs have also demonstrated potential in clinical applications such as survival prediction [22]. Nevertheless, DGMs often suffer from posterior collapse and mode dropping when modeling sparse and high-dimensional clinical tabular data [23]. Normalizing Flows (NF) [24] have therefore been introduced to enhance latent distribution flexibility by transforming simple priors into more expressive distributions [25,26]. Moreover, hybrid approaches such as SMOTified-GAN [27] and SmoteGAN [28] demonstrate that combining heuristic augmentation with generative modeling can improve training stability and generation quality [29].

Despite these advances, iterative generative refinement remains challenging. Recursive training can improve distribution modeling but may also introduce self-reinforcing errors, a phenomenon described as Model Autophagy Disorder (MAD) [30]. Such degradation may gradually reduce diversity and cause generated distributions to deviate from the original data manifold [31,32]. Existing global modeling approaches, including CTGAN [20], TabDDPM [33], and density-based TabKDE [34], generally model the entire distribution without explicitly considering local pathological structures. Therefore, effective iterative generation requires mechanisms that preserve structural information while enabling local distribution refinement.

In this work, we propose **IFC-HFlowVAE** (**I**terative **F**eedback & **C**onsensus framework based on **H**eterogeneous **Flow**-enhanced β-**VAE**). The framework introduces structural anchors into generative training by incorporating SMOTE-generated samples into an augmented reference set, which provides additional guidance for iterative optimization. Furthermore, a GMM-based divide-and-conquer refinement strategy [35] is employed to capture local sub-cluster characteristics. This design is motivated by the intuition that localized modeling can reduce distribution complexity within homogeneous regions [36], and its effectiveness is evaluated empirically through fidelity assessment and downstream classification performance.

The main contributions of this work are summarized as follows:

1. **Framework Novelty:** We propose the IFC-HFlowVAE framework, which integrates heuristic structural guidance with deep generative modeling to alleviate structural overfitting and improve minority-class representation.
2. **Stability Mechanism:** We introduce an augmented reference set and a Weight-Progressive Feedback Loop to stabilize iterative training and reduce distribution degradation during recursive refinement.
3. **Heterogeneous HFlow-VAE:** We develop a heterogeneous Flow-enhanced β-VAE architecture that improves latent distribution modeling while maintaining consistency between numerical and categorical attributes in medical tabular data.
4. **Local Refinement:** We incorporate a GMM-based refinement strategy to model local pathological sub-clusters and enhance downstream classification performance through more representative synthetic samples.

## Materials and methods

### Clinical datasets and preprocessing

Seven clinical tabular datasets were used to evaluate IFC-HFlowVAE: Breast Cancer Coimbra (BCC), PIMA Diabetes (PIMA, see S1 Dataset), TCGA-InfoWithGrade (TCGA), Thyroid Differentiation (THY), Diabetes Risk Prediction (DRP), Framingham (FRM), and Hepatitis (HEP). The datasets were collected from the UCI Machine Learning Repository (BCC, TCGA, THY, DRP, and HEP) and Kaggle (PIMA and FRM).

The selected datasets cover diverse clinical tabular characteristics, including different attribute compositions, sample sizes, feature dimensions, and imbalance ratios. According to their dominant attribute types, datasets are categorized as numerical, categorical, or mixed-type, with detailed statistics summarized in Table 1. In all experiments, the proposed oversampling strategy targets the patient class for synthetic sample generation, regardless of its original class proportion.

**Table 1:** Dataset summary statistics.

Dataset	Samples	Features	n_num	n_cat	IR	Type
BCC	116	9	9	0	0.81	Numerical
PIMA	768	8	8	0	1.87	Numerical
TCGA	839	23	1	22	1.38	Categorical
THY	383	16	1	15	2.55	Categorical
DRP	520	16	1	15	0.63	Categorical
FRM	4,240	15	8	7	5.58	Mixed-type
HEP	155	19	6	13	3.84	Mixed-type

### The IFC iterative feedback framework

**Fig 1:**
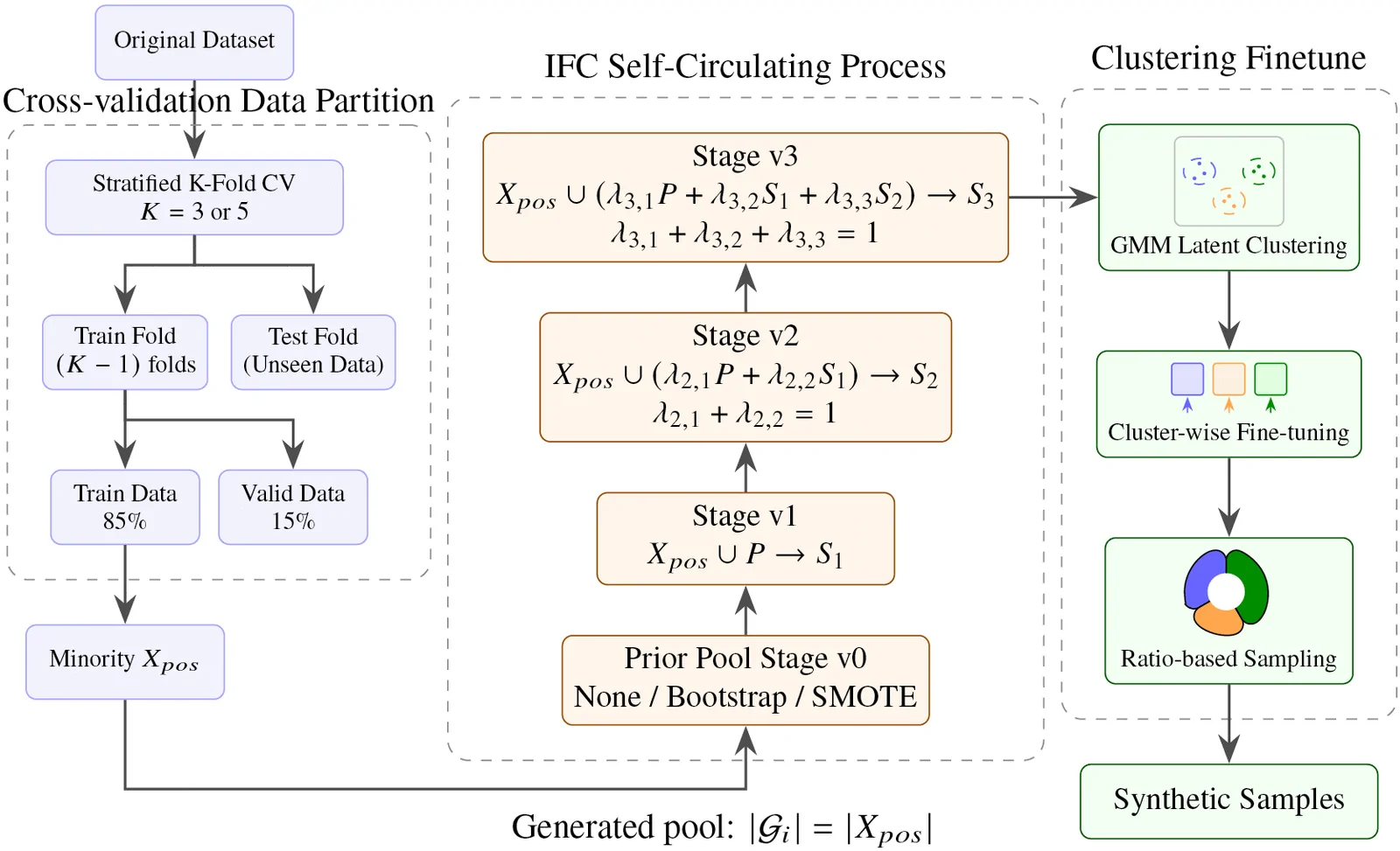
Architecture of proposed IFC-HFlowVAE framework.

The IFC framework generates synthetic minority-class samples for imbalanced clinical tabular data through an iterative feedback process. As illustrated in Fig 1, the framework consists of two sequential components: a self-circulating generation process based on a growing reference pool and a cluster-aware refinement process for localized sampling.

Given the minority training set $X_{pos}$, IFC first constructs an initial prior pool *P* using either bootstrap resampling or SMOTE-based interpolation. This prior pool provides the starting reference samples for HFlowVAE training. The generator then performs multiple self-circulating stages, where each stage combines the original minority samples with weighted samples from the accumulated generation pool. The newly generated samples are added back into this pool and used as references for subsequent stages. In this work, three generation stages are explored, producing intermediate synthetic sets *S*_1_, *S*_2_, and *S*_3_.

After the global generation process, IFC applies a cluster-aware refinement procedure. The latent representations of minority samples are partitioned using Gaussian Mixture Model (GMM) clustering, and the generator is fine-tuned within each cluster to model local characteristics. Finally, synthetic samples are generated from each refined cluster according to the corresponding cluster proportions.

The following subsections provide detailed descriptions of the self-circulating feedback evolution and cluster-aware localized refinement strategies.

#### Self-circulating feedback evolution.

The self-circulating process shown in Fig 1 is designed to iteratively update the reference pool and improve the generated sample quality through multiple training stages. Given the minority training set $X_{pos}$, the required number of synthetic samples $N_{syn}$ is adaptively determined according to the imbalance ratio ($IR=n_{neg}/n_{pos}$):


$$\begin{array}{c} N_{syn}=\left\{ \begin{array}{cc} n_{neg}-n_{pos}, & IR>2,\\ n_{pos}, & IR\leq 2. \end{array}\right. \end{array}$$ (1)

This strategy achieves approximate class balancing under severe imbalance while avoiding excessive synthetic expansion when the original class distribution is relatively balanced.

The evolution process begins with the construction of an initial prior pool *P* from the minority samples. Depending on the selected prior type, *P* is generated using either bootstrap resampling or SMOTE-based interpolation. This prior pool provides a structure-preserving initialization that guides the first-stage HFlowVAE training toward the support region of the observed minority distribution.

During each iterative stage, the training set is constructed by combining the original minority samples with a weighted subset of the accumulated candidate pool:


$$\begin{array}{c} D_{i}=X_{pos}\cup \text{WeightedSample}(𝒢,\lambda_{i};N_{syn}), \end{array}$$ (2)

where $𝒢=[P,S_{1},...,S_{i-1}]$ denotes the growing reference pool containing the prior samples and outputs from previous stages. The weight vector $\lambda_{i}$ controls the contribution of different generations at stage *i*. After training, the newly generated samples $S_{i}$ are appended to 𝒢 and used as additional references in subsequent iterations.

Through this self-circulating mechanism, IFC gradually shifts the learning focus from heuristic initialization toward the non-linear manifold captured by HFlowVAE while maintaining consistency with the original minority distribution. In this study, the framework is instantiated with three iterative stages ($v_{1},v_{2},v_{3}$). The complete procedure is described in Self-Circulating Generation Algorithm 1.

**Algorithm 1 IFC Self-Circulating Generation (General *T*-Stage Form)**

**Require:** Real minority training set $X_{pos}$ ($|X_{pos}|=N$), validation set $X_{val}$, prior type τ∈{bootstrap,smote}, number of stages *T*, weight schedule $\{\lambda_{i}{\}}_{i=1}^{T}$, where $\lambda_{i}\in \Delta^{i-1}$ is an *i*-dimensional probability vector (i.e., $\sum_{j}\lambda_{i,j}=1$)

**Ensure:** Final-stage generator $\theta_{T}$

1: $P\leftarrow \text{MakePriorPool}(X_{pos},\tau ,N)$        ▷ resampling or SMOTE, |*P*|=*N*

2: 𝒢←[P]                 ▷ growing pool of candidate sources, index 0

3: **for** *i* = 1 **to** *T* **do**

4:   $D_{i}\leftarrow X_{pos}\cup \text{WeightedSample}(𝒢,\lambda_{i};\;N)$       ▷ $\lambda_{i}$ has |𝒢|=i components summing to 1

5:   $\theta_{i}\leftarrow \text{TrainVAE}(D_{i},X_{val}\;[,\text{ warm\_start}=\theta_{i-1}])$    ▷ Train HFlowVAE

6:    $S_{i}\leftarrow \text{Generate}(\theta_{i},N)$

7:    $𝒢\leftarrow 𝒢\;\|\;[S_{i}]$       ▷ append this stage’s output to the pool

8: **end for**

9: **return** $\theta_{T}$

#### Cluster-aware localized refinement.

After global self-circulating generation, IFC performs cluster-aware refinement to preserve local minority structures, as illustrated in Fig 1. The encoder of the pre-trained HFlowVAE is first used to obtain latent representations of minority samples, which are then partitioned into *K* clusters using a Gaussian Mixture Model (GMM). Each cluster $C_{k}$ represents a local minority sub-distribution, enabling IFC to capture heterogeneous feature combinations that may be smoothed during global generation.

For each cluster, the global HFlowVAE is fine-tuned using cluster-specific samples with warm-start initialization:


$$\begin{array}{c} \theta_{k}=\text{TrainVAE}(C_{k},C_{k}^{val};\text{warm\_start}=\theta_{base}). \end{array}$$ (3)

The refined cluster-specific generators subsequently generate synthetic samples according to the proportion of each cluster, and all generated subsets are aggregated as the final synthetic minority set. This localized refinement strategy allows IFC to maintain global distribution consistency while enhancing the representation of minority substructures. The detailed procedure is provided in Cluster-Aware Fine-Tuning Algorithm 2.

**Algorithm 2 Cluster-Aware Fine-Tuning and Ratio-Based Sampling**

**Require:** Base generator $\theta_{\text{base}}$, $X_{pos}$, $X_{val}$, max clusters $K_{\max}$, target synthetic count *n*_final_

**Ensure:** Synthetic minority set *X*_syn_

1: $Z\leftarrow {\text{Encode}}_{\theta_{\text{base}}}(X_{pos})$▷ latent means μ(x)

2: $K\leftarrow \min\;(K_{\max},\;\lfloor |X_{pos}|/4\rfloor )$

3: **if** K≤1 **then**

4:  **return** $\text{Generate}(\theta_{\text{base}},n_{\text{final}})$▷ skip fine-tuning

5: **end if**

6: $\left\{C_{1},...,C_{K}\},\text{GMM}\leftarrow \text{FitGMM}(Z,K\right)$▷ diagonal covariance

7: $\left\{C_{1}^{val},...,C_{K}^{val}\}\leftarrow \text{AssignClusters}(X_{val},\text{GMM}\right)$

8: **for** *k* = 1 **to** *K* **do**

9:  $n_{k}\leftarrow \text{round}(n_{\text{final}}\cdot |C_{k}|/|X_{pos}|)$

10:  $\theta_{k}\leftarrow \text{TrainVAE}(C_{k},C_{k}^{val};\text{ warm\_start}=\theta_{\text{base}})$

11:  $S_{k}\leftarrow \text{Generate}(\theta_{k},n_{k})$

12: **end for**

13: $X_{\text{syn}}\leftarrow {⋃}_{k=1}^{K}S_{k}$

14: **return** *X*_syn_

### HFlowVAE: A Heterogeneous flow-augmented VAE for mixed-type clinical data

**Fig 2:**
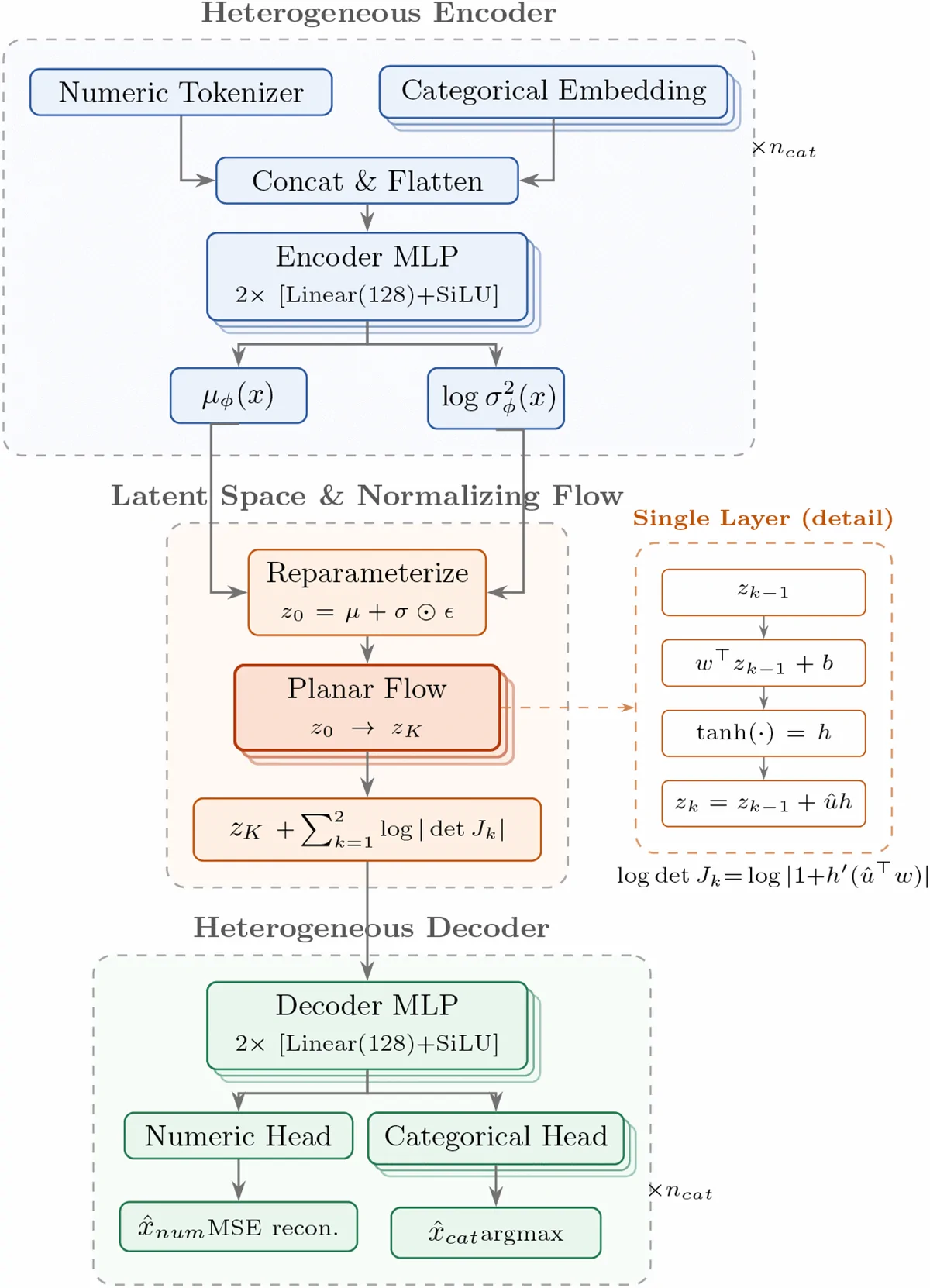
Architecture of HFlowVAE.

HFlowVAE is a heterogeneous variational autoencoder designed for mixed-type clinical tabular data. It integrates attribute-specific encoding and decoding with Normalizing Flow-based latent refinement to improve the flexibility of latent representation learning. The overall architecture of HFlowVAE is illustrated in Fig 2.

**Heterogeneous encoding and decoding.** Numerical and categorical attributes are processed through separate encoding branches. Numerical variables are transformed using a learnable tokenizer, while categorical variables are mapped into embedding representations. The resulting representations are concatenated and fed into an encoder MLP to obtain the posterior parameters $\mu_{\phi }(x)$ and $\log\sigma_{\phi }^{2}(x)$.

The decoder reconstructs different attribute types using dedicated output heads. Numerical attributes are recovered through a numerical reconstruction head, while categorical attributes are reconstructed through categorical classification heads. The corresponding reconstruction losses are optimized separately according to attribute types.

**Normalizing Flow latent refinement.** To relax the Gaussian posterior assumption of standard VAE, HFlowVAE applies Planar Normalizing Flow [37] after the reparameterization step. The latent variable is iteratively transformed as:


$$\begin{array}{c} z_{k}=z_{k-1}+u_{k}\tanh(w_{k}^{T}z_{k-1}+b_{k}), \end{array}$$ (4)

where $u_{k}$, $w_{k}$, and $b_{k}$ are learnable parameters. The Jacobian correction term is incorporated into the variational objective:


$$\begin{array}{c} \log|\det J_{k}|=\log|1+h^{\prime }({\hat{u}}_{k}^{T}z_{k-1}){\hat{u}}_{k}^{T}w_{k}|. \end{array}$$ (5)

The resulting flow-based latent distribution is used in the variational objective.

**Training objective.** The training objective jointly optimizes heterogeneous attribute reconstruction and latent regularization. Numerical and categorical attributes are reconstructed using mean squared error (MSE) and cross-entropy losses, respectively. To automatically balance the contribution of different attribute types, the reconstruction weights are learned through homoscedastic uncertainty weighting. The final objective is formulated as:


$$\begin{array}{c} ℒ=\lambda_{num}{ℒ}_{num}+\lambda_{cat}{ℒ}_{cat}+\beta {ℒ}_{KL}, \end{array}$$ (6)

where $\lambda_{num}$ and $\lambda_{cat}$ denote adaptive weights derived from homoscedastic uncertainty estimation, and β controls the strength of latent regularization. ${ℒ}_{KL}$ represents the KL divergence between the flow-enhanced posterior distribution and the prior distribution.

### Experimental setup

#### Baseline methods and classifiers.

IFC-HFlowVAE is compared with representative baselines covering diverse generative paradigms for tabular data synthesis, including interpolation-based oversampling (SMOTE [13]), deep generative models (CTGAN and TVAE [20]), diffusion-based approaches (TabDDPM [33] and Forest Diffusion [38]), and non-parametric density estimation (TabKDE [34]). These methods provide comprehensive comparisons across classical augmentation, neural generative modeling, diffusion processes, and statistical distribution estimation. A summary of the baseline methods and their corresponding generation paradigms is provided in Table 2.

**Table 2:** Overview of baseline methods and their generation paradigms.

Category	Method	Generation paradigm
Oversampling	SMOTE	Interpolation-based augmentation
Deep generative model	CTGAN	Conditional adversarial generation
Deep generative model	TVAE	Variational autoencoder-based generation
Diffusion model	ForestDiff	Tree-guided diffusion generation
Diffusion model	TabDDPM	Diffusion probabilistic modeling
Density estimation	TabKDE	Kernel density estimation

The robust utility of the resulting synthetic samples is verified using three representative classifiers: Linear Support Vector Classifier (LinearSVC), Multi-Layer Perceptron (**MLP**), and extreme gradient boosting(XGBoost). These classifiers represent kernel-based, neural-based, and ensemble-based learning paradigms, respectively, allowing us to evaluate the classifier-agnostic quality of the generated manifolds. Due to space constraints, classifier names are abbreviated in the result tables, where **SVC** refers to LinearSVC and **XGB** refers to XGBoost.

#### Implementation details.

The base HFlowVAE hyperparameters (token/latent dim = 16, hidden widths = 128/128, linear numeric tokenizer, planar flow with K = 2 and KL correction enabled, β=0.3 with 50 warmup steps and free bits = 0.02, batch size = 64, homoscedastic uncertainty weighting, quantile transform, and ordinal encoding) were fixed. Only the changes shown in Table 3 were applied during fine-tuning.

**Table 3:** Comparison of changed base and fine-tuned hyperparameters.

Hyperparameter	Base value	Fine-tune value
Learning rate	$1\times 10^{-3}$	$1\times 10^{-4}$
Patience	50	20
Max epochs	1,000	500
$K_{\max}$ (new)	N/A	5
Min samples/cluster (new)	N/A	4

We employed stratified *K*-fold cross-validation, with *K* = 3 for datasets with fewer than 300 samples and *K* = 5 otherwise, repeated over five random seeds (42, 456, 1024, 2026, 3407). For each fold, one fold was held out as the test set, and the remaining K−1 folds were further split into training (85%) and validation (15%) sets. All preprocessing steps were fitted exclusively on the training split of each fold to prevent data leakage. Early stopping was monitored on the validation set with a progress window of 5 epochs and a minimum improvement $\delta =1\times 10^{-4}$; if the validation set contained fewer than 5 samples, a fallback threshold was applied. The PQ threshold α was set to 1.0 and the EMA rate γ to 0.3.

The self-circulating process consists of three progressive stages (v1–v3) as illustrated in Fig 1. At each stage, the weight vector $\lambda_{i}$ determines the contribution of the original minority set and previously generated synthetic subsets in the augmented reference set 𝒢. Two weight configurations are instantiated in this study and are denoted as Schedule-I and Schedule-II, respectively (Table 4).

**Table 4:** Instantiations of the self-circulating schedule used in this paper.

Stage	Weight vector $\lambda_{i}$ over $𝒢=[P,S_{1},...,S_{i-1}]$	
	case 1	case 2
v1	(1.0)	(1.0)
v2	(0.3, 0.7)	(0.5, 0.5)
v3	(0.2, 0.3, 0.5)	(0.5, 0.3, 0.2)

**Classification models.** All classifiers were trained on identical training splits and evaluated on the corresponding test sets; their main hyperparameters are summarized in Table 5.

**Table 5:** Configurations of downstream classifiers.

Classifier	Key settings
SVC	LinearSVC, *C* = 0.05, class_weight = balanced, max_iter = 1000
MLP	$hidden_{l}ayer_{s}izes=(64,32)$, α=0.01, early stopping, validation fraction = 0.15, max_iter = 1000
XGB	$n_{estimators}=100$, max depth = 2, learning rate = 0.05, subsample = 0.8, colsample_bytree = 0.8

#### Evaluation metrics.

Downstream classification performance is evaluated using F1-score, G-mean, and Recall, averaged over 5-fold cross-validation with 5 random seeds (3-fold cross-validation for BCC and HEP due to limited sample sizes).

To evaluate distributional fidelity, we assess whether synthetic data preserve important characteristics of marginal distributions and attribute dependencies.

**Skewness.** For numerical attributes, the bias-corrected Fisher–Pearson skewness coefficient is calculated to measure whether the asymmetry of real attribute distributions is preserved in synthetic data.

**Multimodality.** For numerical attributes, Gaussian kernel density estimation (KDE) with Silverman’s rule for bandwidth selection is applied to identify local density peaks. The number and locations of detected modes are compared between real and synthetic data to evaluate whether latent sub-population structures are preserved.

**Correlation/Association Absolute Deviation (CAD).** To evaluate dependency preservation in heterogeneous tabular data, we propose CAD, which aggregates multiple association measures according to attribute types. Specifically, Kendall’s rank correlation coefficient, Cramér’s V, and correlation ratio are used for numerical–numerical, categorical–categorical, and numerical–categorical attribute pairs, respectively. Given the resulting association matrices of real and synthetic datasets ($A^{real}$ and $A^{syn}$), CAD is defined as:


$$\begin{array}{c} CAD=\frac{2}{d(d-1)}\sum_{i<j}\left|A_{ij}^{real}-A_{ij}^{syn}\right|, \end{array}$$ (7)

where *d* denotes the number of attributes. Lower CAD values indicate better preservation of attribute dependency structures.

## Results and discussion

### Baseline performance

The classification difficulty of the evaluated datasets is reflected by the baseline results without augmentation (Table 6). For compact presentation, leading zeros are omitted in all reported mean and standard deviation values throughout the following tables. Datasets such as DRP, THY, and TCGA exhibit relatively high baseline performance across different classifiers, suggesting clear class separability and less severe distributional ambiguity. In contrast, datasets with limited sample sizes or more complex feature interactions present greater classification challenges.

**Table 6:** Performance of baseline classification results (Mean ± Std).

Dataset	SVC		MLP		XGB	
	F1-score	G-mean	F1-score	G-mean	F1-score	G-mean
BCC	.70 ± .08	.67 ± .08	.56 ± .19	.50 ± .13	.73 ± .05	.70 ± .08
PIMA	.67 ± .04	.74 ± .03	.57 ± .09	.66 ± .07	.63 ± .05	.71 ± .04
TCGA	.86 ± .03	.88 ± .03	.83 ± .02	.86 ± .02	.85 ± .02	.87 ± .02
THY	.91 ± .03	.94 ± .03	.81 ± .08	.85 ± .07	.93 ± .04	.94 ± .04
DRP	.93 ± .03	.92 ± .03	.92 ± .03	.90 ± .05	.94 ± .02	.92 ± .02
FRM	.38 ± .02	.67 ± .02	.06 ± .05	.15 ± .11	.06 ± .03	.17 ± .05
HEP	.59 ± .11	.75 ± .09	.28 ± .22	.39 ± .24	.44 ± .14	.58 ± .12

Notably, FRM remains the most challenging dataset, where the MLP and XGB classifiers achieve extremely low F1-scores (0.06±.05 and 0.06±.03) and G-mean values (0.15±.11 and 0.17±.05), indicating substantial difficulty in recognizing minority-class patterns. Although SVC achieves a higher G-mean (0.67±.02), its low F1-score (0.38±.02) still reflects limited minority-class predictive capability. These results suggest that such datasets suffer from severe class overlap and insufficient minority representation, highlighting the need for effective distribution reconstruction beyond conventional classifier optimization.

### Classification performance

**Numerical datasets.** The classification results on numerical datasets are summarized in Table 7. These datasets generally exhibit relatively stable classification performance, indicating that numerical attributes provide informative representations for distinguishing minority and majority classes. For datasets with relatively clear decision boundaries, such as TCGA and THY, most augmentation methods achieve comparable performance, suggesting that the potential improvement from synthetic data is limited when the original feature space already provides sufficient discriminative information.

**Table 7:** Classification performance comparison on numerical datasets using different augmentation methods (Mean ± Std).

Dataset	Method	SVC		MLP		XGB	
		F1-score	G-mean	F1-score	G-mean	F1-score	G-mean
BCC	SMOTE	**.70 ± .07**	.67 ± .08	.69 ± .07	.42 ± .17	.78 ± .06	.70 ± .09
	CTGAN	.60 ± .13	.61 ± .10	.69 ± .08	.36 ± .21	.76 ± .04	.68 ± .07
	TVAE	.69 ± .07	.67 ± .07	.69 ± .06	.48 ± .18	**.78 ± .07**	.70 ± .10
	ForestDiff	.62 ± .10	.61 ± .09	.70 ± .05	.34 ± .20	.73 ± .05	.66 ± .07
	TabDDPM	.66 ± .08	.65 ± .08	.69 ± .06	.40 ± .18	.75 ± .07	.67 ± .10
	TabKDE	.70 ± .08	**.68 ± .07**	.69 ± .06	.40 ± .18	.75 ± .05	.67 ± .07
	HFlowVAE-only	.70 ± .08	.67 ± .09	.69 ± .08	**.50 ± .16**	.77 ± .07	**.71 ± .09**
	IFC	.69 ± .08	.67 ± .08	**.70 ± .07**	.48 ± .17	.77 ± .06	.71 ± .09
PIMA	SMOTE	.67 ± .04	.74 ± .03	.66 ± .05	.73 ± .05	**.69 ± .03**	**.76 ± .03**
	CTGAN	.64 ± .05	.72 ± .04	.62 ± .03	.70 ± .03	.65 ± .04	.72 ± .04
	TVAE	.66 ± .03	.74 ± .02	.65 ± .04	.72 ± .04	.67 ± .04	.74 ± .03
	ForestDiff	.61 ± .05	.69 ± .04	.62 ± .04	.70 ± .04	.62 ± .04	.70 ± .03
	TabDDPM	.67 ± .04	.74 ± .03	.65 ± .04	.72 ± .04	.68 ± .04	.75 ± .03
	TabKDE	**.67 ± .04**	**.75 ± .04**	**.67 ± .04**	**.74 ± .04**	.68 ± .04	.75 ± .03
	HFlowVAE-only	.67 ± .05	.74 ± .04	.65 ± .05	.72 ± .05	.68 ± .05	.75 ± .04
	IFC	.66 ± .05	.73 ± .04	.64 ± .04	.71 ± .04	.67 ± .05	.74 ± .04

Note: Bold indicates the best-performing result, underlined denotes the second best.

In cases where more than two ties occur, no specific formatting is applied.

For more challenging cases, such as BCC and PIMA, the impact of augmentation becomes more evident. Different generative approaches show varying degrees of improvement depending on the classifier, reflecting the dependency between synthetic data quality and downstream learning algorithms. IFC-HFlowVAE achieves competitive performance across these datasets, maintaining stable F1-score and G-mean values compared with existing augmentation methods. In particular, the consistent performance across different classifiers indicates that the proposed framework can generate minority samples with sufficient diversity while preserving useful class-related structures.

**Categorical datasets.** The results on categorical datasets are presented in Table 8. Compared with purely numerical datasets, categorical datasets introduce additional challenges due to discrete feature spaces and sparse combinations of categorical patterns. Therefore, effective augmentation requires not only increasing minority sample quantity but also preserving meaningful categorical dependencies.

**Table 8:** Classification performance comparison on categorical datasets using different augmentation methods (Mean ± Std).

Dataset	Method	SVC		MLP		XGB	
		F1-score	G-mean	F1-score	G-mean	F1-score	G-mean
TCGA	SMOTE	.86 ± .03	.88 ± .02	.85 ± .03	.87 ± .03	**.86 ± .03**	**.88 ± .02**
	CTGAN	.86 ± .03	.88 ± .02	.84 ± .03	.86 ± .03	.85 ± .03	.87 ± .02
	TVAE	.86 ± .03	.88 ± .03	.84 ± .03	.86 ± .03	.86 ± .03	.87 ± .03
	ForestDiff	.83 ± .03	.85 ± .03	.83 ± .03	.85 ± .03	.84 ± .02	.86 ± .02
	TabDDPM	.86 ± .03	.88 ± .03	**.85 ± .03**	**.87 ± .03**	.85 ± .02	.87 ± .02
	TabKDE	.86 ± .03	.88 ± .02	.85 ± .03	.87 ± .03	.86 ± .03	.87 ± .02
	HFlowVAE-only	**.86 ± .03**	**.88 ± .03**	.85 ± .03	.87 ± .03	.86 ± .03	.88 ± .03
	IFC	.86 ± .03	.88 ± .02	.85 ± .03	.87 ± .03	.85 ± .02	.87 ± .02
THY	SMOTE	.92 ± .04	.93 ± .04	**.87 ± .07**	**.90 ± .05**	.93 ± .05	.94 ± .04
	CTGAN	.92 ± .04	.94 ± .03	.83 ± .07	.88 ± .05	.91 ± .04	.94 ± .03
	TVAE	.92 ± .04	.94 ± .03	.85 ± .07	.90 ± .05	**.93 ± .04**	**.95 ± .03**
	ForestDiff	.92 ± .04	.93 ± .04	.82 ± .10	.86 ± .08	.92 ± .04	.94 ± .04
	TabDDPM	.92 ± .03	.94 ± .03	.85 ± .06	.88 ± .06	.92 ± .05	.94 ± .04
	TabKDE	.91 ± .03	.94 ± .03	.86 ± .06	.90 ± .05	.93 ± .04	.95 ± .03
	HFlowVAE-only	.92 ± .04	.94 ± .03	.82 ± .08	.87 ± .07	.93 ± .04	.95 ± .03
	IFC	**.92 ± .04**	**.94 ± .03**	.83 ± .07	.88 ± .05	.93 ± .04	.94 ± .03
DRP	SMOTE	**.93 ± .02**	**.92 ± .02**	**.95 ± .02**	**.92 ± .03**	.94 ± .02	.91 ± .03
	CTGAN	.92 ± .02	.92 ± .02	.93 ± .03	.91 ± .04	.94 ± .02	.91 ± .02
	TVAE	.92 ± .03	.92 ± .03	.94 ± .02	.92 ± .04	**.94 ± .02**	**.91 ± .02**
	ForestDiff	.92 ± .03	.91 ± .03	.94 ± .03	.91 ± .04	.94 ± .02	.91 ± .03
	TabDDPM	.93 ± .02	.92 ± .03	.94 ± .02	.92 ± .04	.94 ± .02	.91 ± .03
	TabKDE	.93 ± .03	.92 ± .03	.94 ± .02	.92 ± .03	.94 ± .02	.91 ± .03
	HFlowVAE-only	.92 ± .03	.92 ± .03	.94 ± .03	.92 ± .04	.94 ± .02	.91 ± .03
	IFC	.93 ± .03	.92 ± .03	.94 ± .02	.91 ± .04	.94 ± .02	.91 ± .03

Note: Bold indicates the best-performing result, underlined denotes the second best.

In cases where more than two ties occur, no specific formatting is applied.

Across the evaluated categorical datasets, the performance differences among generative methods are relatively dataset-dependent. For datasets with relatively distinguishable class structures, most methods achieve similar classification performance, whereas more difficult datasets exhibit larger variations among augmentation strategies. IFC-HFlowVAE provides competitive results across different classifiers, demonstrating that the heterogeneous latent modeling strategy can capture useful categorical representations without causing severe distributional distortion. These results support the applicability of the proposed framework for medical tabular data containing discrete clinical variables.

**Mixed-type datasets.** Mixed-type datasets represent the most challenging scenario because they require simultaneous modeling of numerical distributions and categorical dependencies. The classification results are reported in Table 9. Compared with single-type datasets, the performance differences among augmentation methods become more apparent, highlighting the difficulty of learning heterogeneous clinical distributions.

**Table 9:** Classification performance comparison on mixed-type datasets using different augmentation methods (Mean ± Std).

Dataset	Method	SVC		MLP		XGB	
		F1-score	G-mean	F1-score	G-mean	F1-score	G-mean
FRM	SMOTE	.34 ± .03	.63 ± .03	.28 ± .03	.53 ± .03	.35 ± .02	.63 ± .02
	CTGAN	.30 ± .04	.52 ± .05	.23 ± .05	.40 ± .05	.26 ± .05	.45 ± .06
	TVAE	.36 ± .02	.65 ± .02	.27 ± .03	.51 ± .04	.28 ± .03	.53 ± .04
	ForestDiff	.29 ± .04	.48 ± .04	.20 ± .03	.36 ± .03	.15 ± .04	.30 ± .04
	TabDDPM	.35 ± .02	.63 ± .02	**.34 ± .03**	.60 ± .03	.36 ± .03	.61 ± .04
	TabKDE	**.37 ± .02**	**.66 ± .02**	.34 ± .02	**.62 ± .02**	**.36 ± .02**	**.65 ± .02**
	HFlowVAE-only	.34 ± .03	.62 ± .03	.28 ± .03	.53 ± .04	.34 ± .03	.62 ± .03
	IFC	.34 ± .03	.61 ± .04	.26 ± .03	.50 ± .03	.33 ± .03	.61 ± .03
HEP	SMOTE	.61 ± .13	.75 ± .10	**.63 ± .07**	**.79 ± .06**	.55 ± .16	.70 ± .13
	CTGAN	.61 ± .10	.75 ± .08	.55 ± .15	.72 ± .14	**.56 ± .10**	**.73 ± .09**
	TVAE	.63 ± .10	.76 ± .08	.60 ± .10	.77 ± .07	.53 ± .19	.68 ± .17
	ForestDiff	**.65 ± .11**	**.78 ± .09**	.58 ± .09	.76 ± .09	.56 ± .12	.70 ± .10
	TabDDPM	.56 ± .13	.71 ± .10	.56 ± .11	.74 ± .09	.50 ± .11	.64 ± .09
	TabKDE	.58 ± .13	.74 ± .10	.60 ± .07	.78 ± .06	.55 ± .15	.72 ± .12
	HFlowVAE-only	.61 ± .14	.75 ± .11	.58 ± .06	.76 ± .05	.53 ± .15	.69 ± .13
	IFC	.59 ± .13	.73 ± .11	.61 ± .09	.78 ± .07	.54 ± .14	.69 ± .11

Note: Bold indicates the best-performing result, underlined denotes the second best.

In cases where more than two ties occur, no specific formatting is applied.

On FRM, all baseline classifiers show substantial degradation without augmentation, indicating severe minority-class recognition difficulty. Synthetic augmentation improves classification performance to different extents, demonstrating the necessity of reconstructing minority distributions in highly imbalanced clinical settings. Although different generative models achieve the best performance on different classifiers, IFC-HFlowVAE consistently improves upon the non-augmented baseline and remains competitive with existing state-of-the-art methods. Similarly, on HEP, the proposed framework achieves comparable performance with leading approaches across multiple classifiers.

Overall, these results indicate that the effectiveness of synthetic augmentation is strongly influenced by dataset characteristics. Rather than universally maximizing classification scores, IFC-HFlowVAE provides a stable augmentation strategy that preserves informative minority-class structures across heterogeneous medical tabular datasets.

### Ablation and sensitivity analysis

For readability, the six IFC configurations are abbreviated as None-NoFT, None-FT, B-NoFT, B-FT, S-NoFT, and S-FT, corresponding to none_noft, none_ft, bootstrap_noft, bootstrap_ft, smote_noft, and smote_ft, respectively. The two weight configurations defined in Table 4 are denoted as Schedule-I and Schedule-II in the following analysis.

**Effect of prior source and fine-tuning strategies.** The six configurations were evaluated under a 3×2 factorial design (prior source: None/bootstrap/SMOTE; fine-tuning: enabled/disabled) using cross-dataset Friedman mean ranks, where lower ranks indicate better performance (Table 10).

**Table 10:** Cross-dataset Friedman mean ranks of six IFC configurations.

Metric	Schedule	None-NoFT	None-FT	B-FT	B-NoFT	S-FT	S-NoFT
F1-score	Schedule-I	**3.31**	3.42	3.41	3.69	3.52	3.66
G-mean	Schedule-I	**3.22**	3.32	3.49	3.81	3.48	3.67
F1-score	Schedule-II	3.44	3.54	3.49	3.50	**3.32**	3.71
G-mean	Schedule-II	3.36	3.48	3.56	3.67	**3.33**	3.60

Across the four metric-schedule combinations, the overall ranking was: None-NoFT (3.33) < S-FT (3.41) <None-FT (3.44) < B-FT (3.49) < S-NoFT (3.66) < B-NoFT (3.67). The Friedman test showed significant differences for G-mean under both schedules (Schedule-I: $\chi^{2}=12.23$, *p* = 0.032; Schedule-II: $\chi^{2}=13.07$, *p* = 0.023), while no significant differences were observed for F1-score (Schedule-I: *p* = 0.073; Schedule-II: *p* = 0.086). After Nemenyi correction, only one pairwise comparison remained significant: S-FT outperformed B-NoFT in G-mean under Schedule-II (*p* = 0.016).

Overall, None-NoFT achieved the best or near-best ranking, suggesting that the learned generative prior itself provides a strong augmentation capability. In contrast, self-circulation without fine-tuning (B-NoFT and S-NoFT) showed inferior performance, whereas fine-tuned variants remained comparable with the HFlowVAE-only baseline.

**Sensitivity to self-circulation depth.** The influence of self-circulation depth was evaluated by comparing two-stage and three-stage schedules. The Holm-corrected *p*-values are summarized in Table 11. None of the eight configuration-metric-schedule comparisons reached statistical significance after correction. A secondary Wilcoxon analysis pooling all self-circulating configurations showed no significant effect for F1-score under either schedule (Schedule-I: *p* = 0.601; Schedule-II: *p* = 0.072), a marginal effect for Schedule-I/G-mean (*p* = 0.083), and a significant effect for Schedule-II/G-mean (*p* = 0.018). The direction of differences depended on the schedule: stage 3 was favored under Schedule-II, whereas stage 2 was favored under Schedule-I.

**Table 11:** Stage-depth (2 vs. 3) comparison, Holm-corrected *p*-values.

Metric	Schedule	B-FT	B-NoFT	S-FT	S-NoFT
F1-score	Schedule-I	0.084	0.353	0.353	0.206
G-mean	Schedule-I	0.479	0.479	0.107	0.084
F1-score	Schedule-II	1.000	1.000	0.301	0.788
G-mean	Schedule-II	0.878	0.544	0.187	0.279

And the depth trend of SMOTE-based self-circulation is summarized in Table 12. S-NoFT exhibited a significant decreasing trend across all metric-schedule combinations. S-FT showed the same trend under Schedule-I, while maintaining stable performance under Schedule-II, where stage-3 performance slightly recovered compared with stage 2 (F1-score: 0.709 vs. 0.707; G-mean: 0.768 vs. 0.767).

**Table 12:** Stage-depth comparison between two-stage and three-stage schedules with Holm-corrected *p*-values.

Config	Metric	Schedule	d1	d2	d3	*p* (decreasing)
S-NoFT	F1-score	Schedule-I	0.716	0.705	0.702	$1.5\times 10^{-5}$
S-FT	F1-score	Schedule-I	0.716	0.708	0.704	$1.1\times 10^{-4}$
S-NoFT	G-mean	Schedule-I	0.772	0.763	0.761	$1.7\times 10^{-3}$
S-FT	G-mean	Schedule-I	0.772	0.767	0.764	$5.97\times 10^{-4}$
S-NoFT	F1-score	Schedule-II	0.716	0.703	0.703	$1.0\times 10^{-3}$
S-FT	F1-score	Schedule-II	0.716	0.707	0.709	0.061 (n.s.)
S-NoFT	G-mean	Schedule-II	0.772	0.763	0.763	$6.8\times 10^{-3}$
S-FT	G-mean	Schedule-II	0.772	0.767	0.768	0.061 (n.s.)

**Sensitivity to schedule configuration.** The effect of different self-circulating schedules was evaluated by comparing Schedule-I and Schedule-II under different configurations. No comparison reached statistical significance after Holm correction. Among all comparisons, S-FT at stage depth 3 showed the strongest trend toward improvement under Schedule-II, although the differences remained non-significant (F1-score: *p* = 0.087; G-mean: *p* = 0.061). These results suggest that the choice of weight configuration alone does not produce a consistent performance gain, but may influence the effectiveness of fine-tuned SMOTE-based self-circulation.

**Synthesis.** Overall, the ablation and sensitivity analyses demonstrate that each component contributes differently to IFC performance. The VAE-based generative prior alone (None-NoFT) provides a strong baseline and achieves the best or near-best ranking across settings. Self-circulation without fine-tuning tends to reduce performance, particularly as recursive depth increases, indicating that additional refinement requires adaptation of the generator to the evolving reference distribution.

Fine-tuning effectively mitigates this degradation, allowing self-circulating variants to remain comparable with the HFlowVAE-only baseline. Under Schedule-II, S-FT achieved the only statistically confirmed improvement over another configuration by outperforming B-NoFT in G-mean (*p* = 0.016). These results suggest that self-circulation is not beneficial by itself, but requires appropriate fine-tuning and reference-set design to provide stable refinement.

### Fidelity analysis

For all fidelity evaluations, the representative (Seed, Fold) is selected as the one closest to the dataset median in raw-data downstream performance, avoiding favorable fold selection. Fidelity is evaluated from marginal distribution preservation (skewness, peak structure, and categorical JS distance), dependency preservation (Kendall correlation), and manifold consistency, with additional analysis on difficult-to-preserve attributes.

**Marginal distribution fidelity.** Table 13 summarizes five fidelity metrics used to evaluate marginal distribution preservation. Skew, Peak, and JS denote the deviation of skewness, peak mismatch rate, and Jensen–Shannon (JS) distance for numerical and categorical attributes, respectively. To further assess the preservation of challenging minority structures, Flag-Skew and Flag-Peak are calculated on numerical attributes identified as highly skewed or multimodal.

**Table 13:** Cross-dataset summary of fidelity metrics (Mean ± Std). ↓ indicates lower is better and ↑ indicates higher is better.

Method	Skew↓	Peak↓	JS↓	Flag-Skew↓	Flag-Peak↑
TabKDE	0.259±0.128	0.042±0.072	0.060±0.045	0.303±0.164	1.000±0.000
SMOTE	0.275±0.250	0.232±0.360	0.123±0.070	0.521±0.554	0.575±0.371
IFC	0.345±0.266	0.232±0.360	0.100±0.060	0.516±0.500	0.625±0.415
CTGAN	0.479±0.235	0.304±0.384	0.066±0.020	0.707±0.273	0.475±0.379
TVAE	0.687±0.175	0.357±0.353	0.086±0.037	0.913±0.255	0.700±0.209
ForestDiff	0.588±0.383	0.649±0.342	0.199±0.110	0.955±0.476	0.725±0.285
TabDDPM	0.606±0.478	0.677±0.392	0.062±0.042	1.008±0.660	0.500±0.306

For overall fidelity, IFC achieves competitive performance across different attribute distributions. Its skewness deviation and peak mismatch are comparable to SMOTE and substantially lower than most neural generative baselines, indicating improved preservation of non-Gaussian numerical characteristics. Although TabKDE achieves the lowest numerical deviations and JS distance remains slightly better for several density-based or neural baselines, IFC maintains a balanced fidelity across numerical and categorical attributes, with a lower overall JS distance than SMOTE.

For challenging numerical attributes, IFC obtains a Flag-Skew score of 0.516 and a Flag-Peak score of 0.625, ranking closely with SMOTE (0.521 and 0.575) and substantially outperforming most neural generative methods. Only TabKDE achieves better preservation on these difficult attributes. These results suggest that IFC is capable of maintaining skewed and multimodal minority structures that are prone to being smoothed during generative modeling.

**Fig 3:**
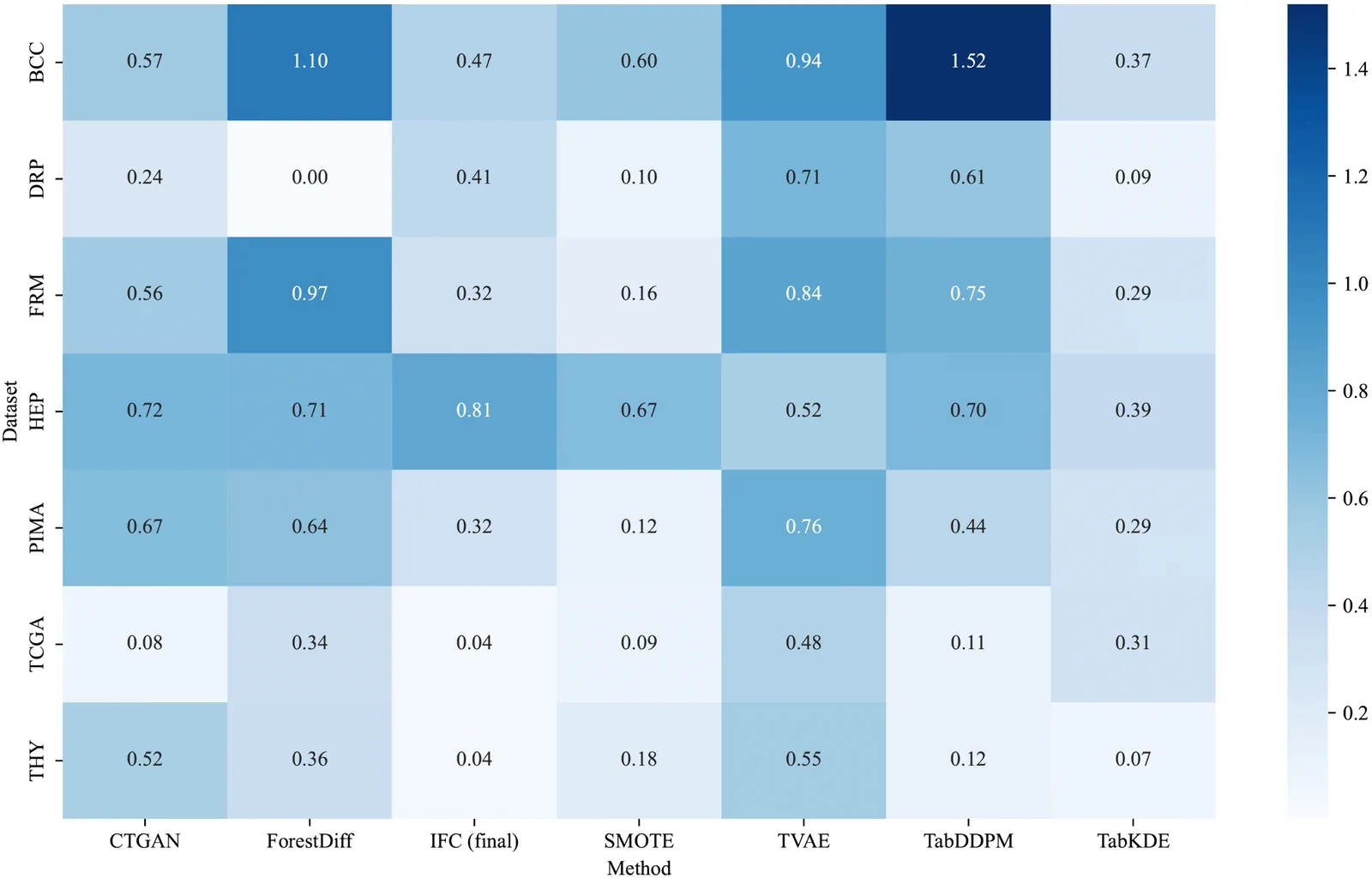
Mean absolute skewness deviation between real and synthetic minority-class samples across datasets. Lower values indicate better fidelity.

Fig 3 further presents the dataset-level skewness deviation comparison, where IFC achieves the best performance on TCGA and THY while maintaining competitive results on other datasets.

**Fig 4:**
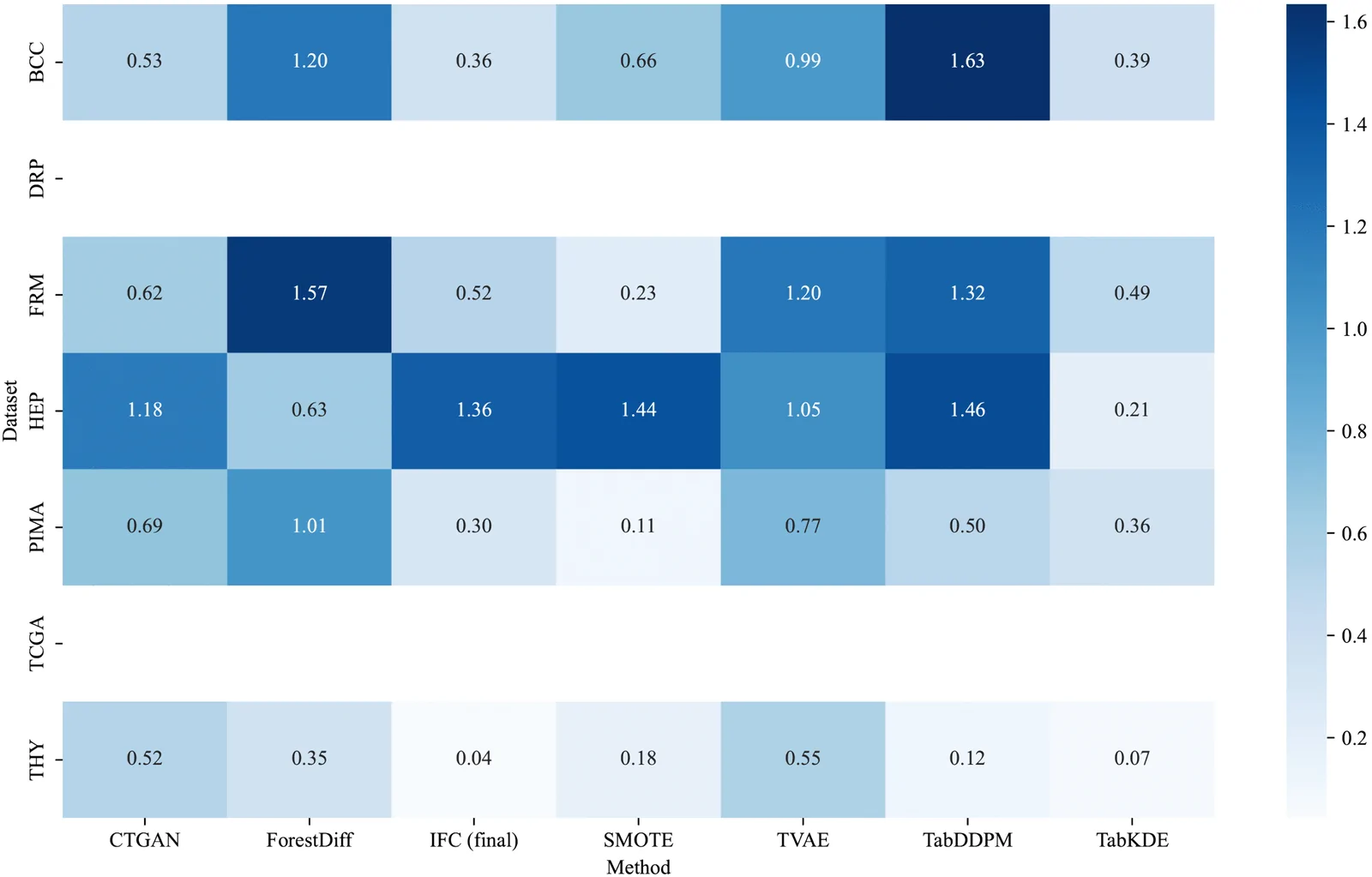
Mean absolute skewness deviation restricted to highly skewed or multimodal numerical attributes. Lower values indicate better preservation of difficult numerical characteristics.

Fig 4 shows the corresponding comparison for difficult numerical attributes. IFC achieves competitive performance on several datasets, including BCC, PIMA, and Thyroid, where categorical distributions are strongly imbalanced. These results indicate that IFC maintains minority-class distribution characteristics beyond simple marginal approximation.

**Fig 5:**
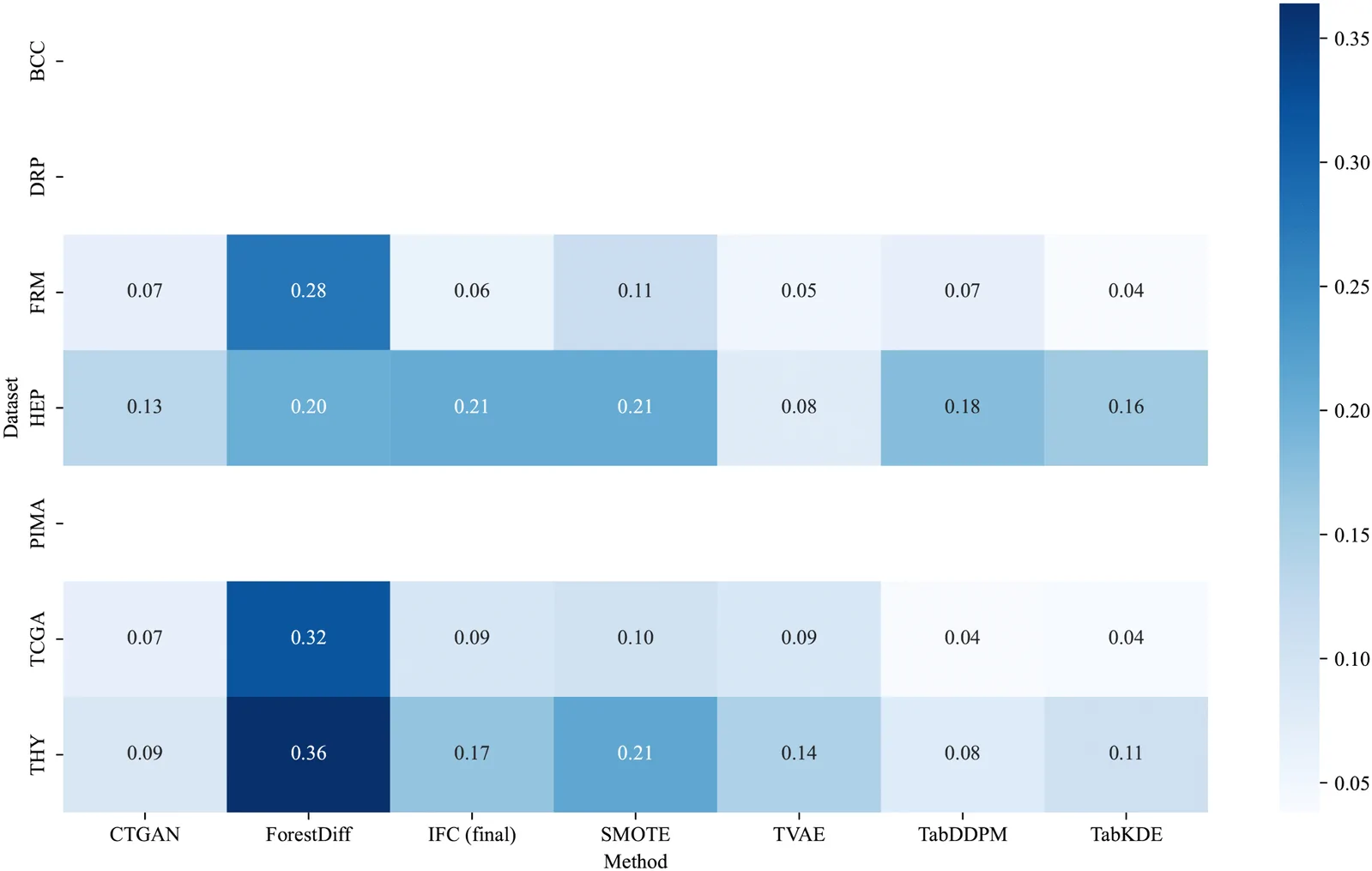
Mean Jensen–Shannon distance between real and synthetic categorical distributions restricted to substantially non-uniform attributes.

Fig 5 presents the fidelity comparison for substantially non-uniform categorical attributes. On FRM, IFC ranks third among all evaluated methods, following TabKDE and TVAE. However, on HEP, TCGA, and THY, the fidelity of IFC remains lower than most competing approaches, with performance only exceeding ForestDiff and SMOTE. This limitation is mainly observed in categorical attributes with extremely imbalanced distributions (e.g., fewer than 1% positive instances in binary attributes). In such cases, the missing categorical patterns cannot be fully recovered through latent refinement, limiting the ability of IFC to preserve rare discrete events.

**Fig 6:**
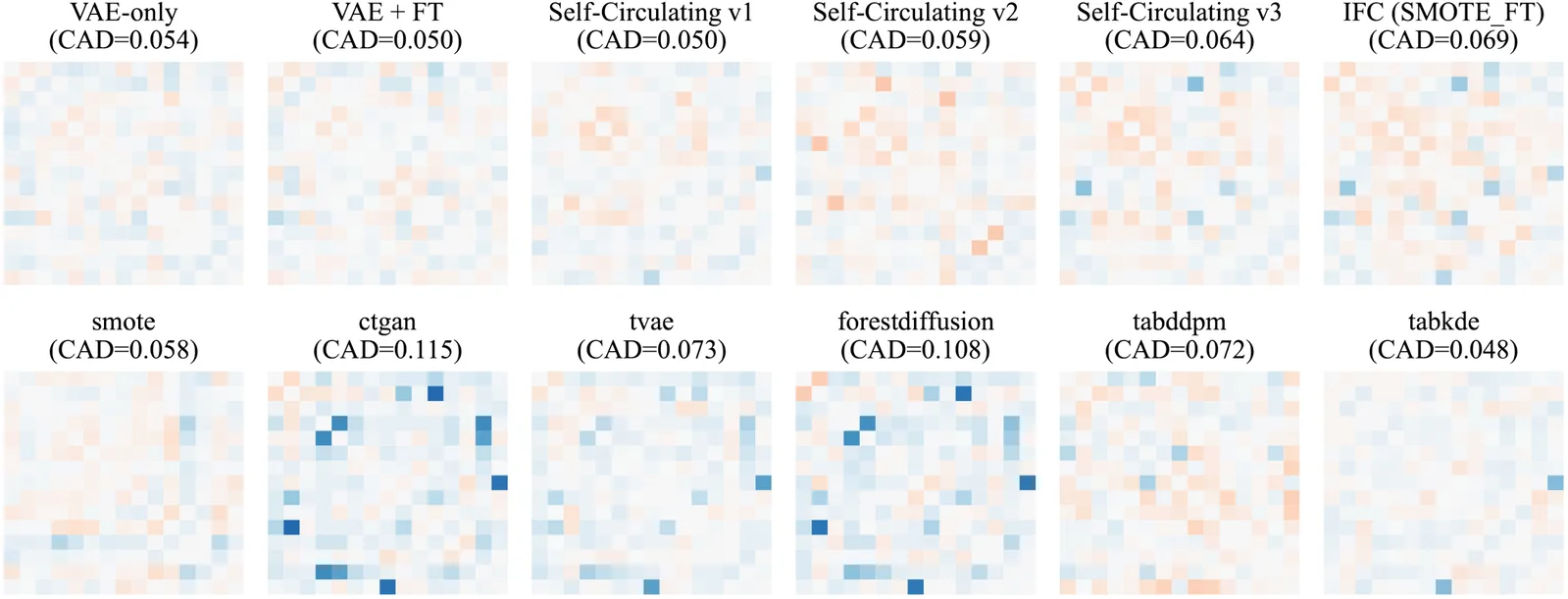
Kendall correlation deviation between synthetic and real samples on FRM.

**Fig 7:**
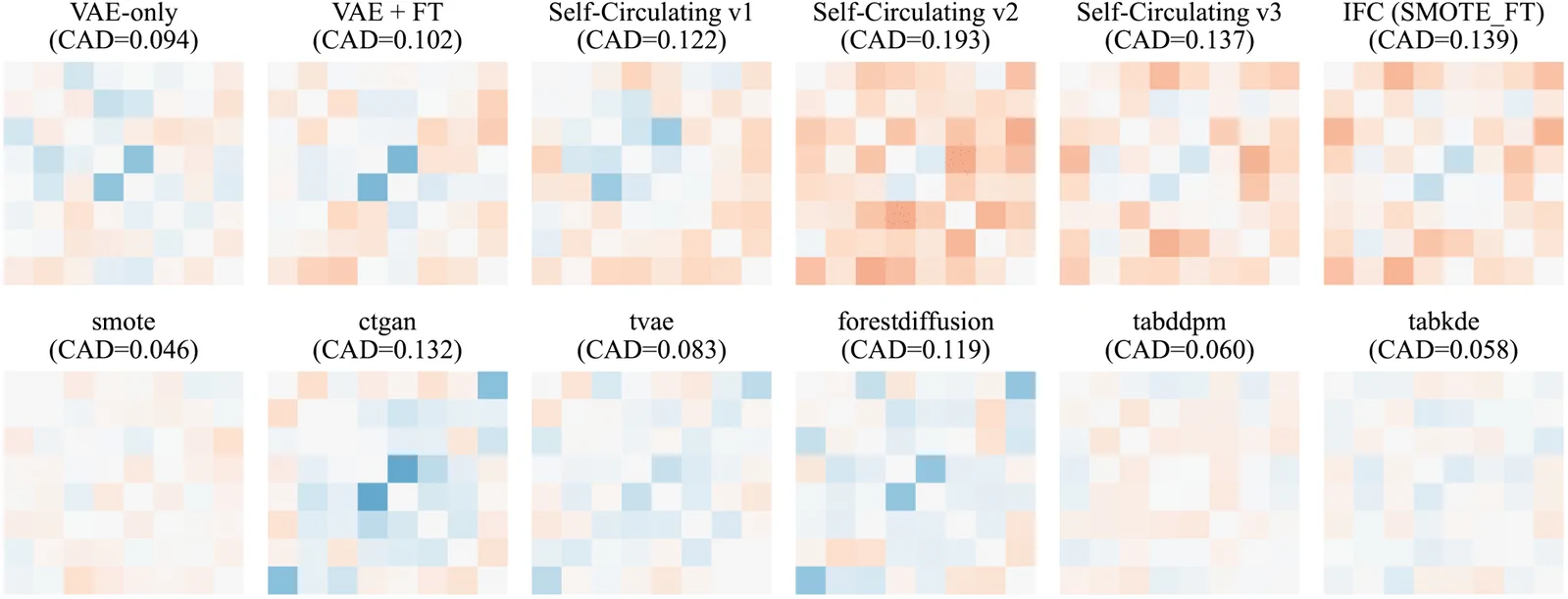
Kendall correlation deviation between synthetic and real samples on PIMA.

**Dependency preservation.** To evaluate whether synthetic samples maintain feature-level dependencies, we analyze Kendall correlation differences on FRM and PIMA, two representative datasets with challenging distributions. Figs 6 and 7 compare Kendall correlation deviations between synthetic and real minority-class samples. Lighter regions indicate closer agreement with real correlations, and CAD values summarize the overall correlation deviation.

Across both datasets, IFC gradually improves dependency preservation during the generation process, with the self-circulating v2 stage generally achieving the closest correlation structure among its variants. This trend is consistent with the downstream classification results. TabKDE and SMOTE provide strong dependency preservation, while CTGAN and TabDDPM show larger deviations.

**Fig 8:**
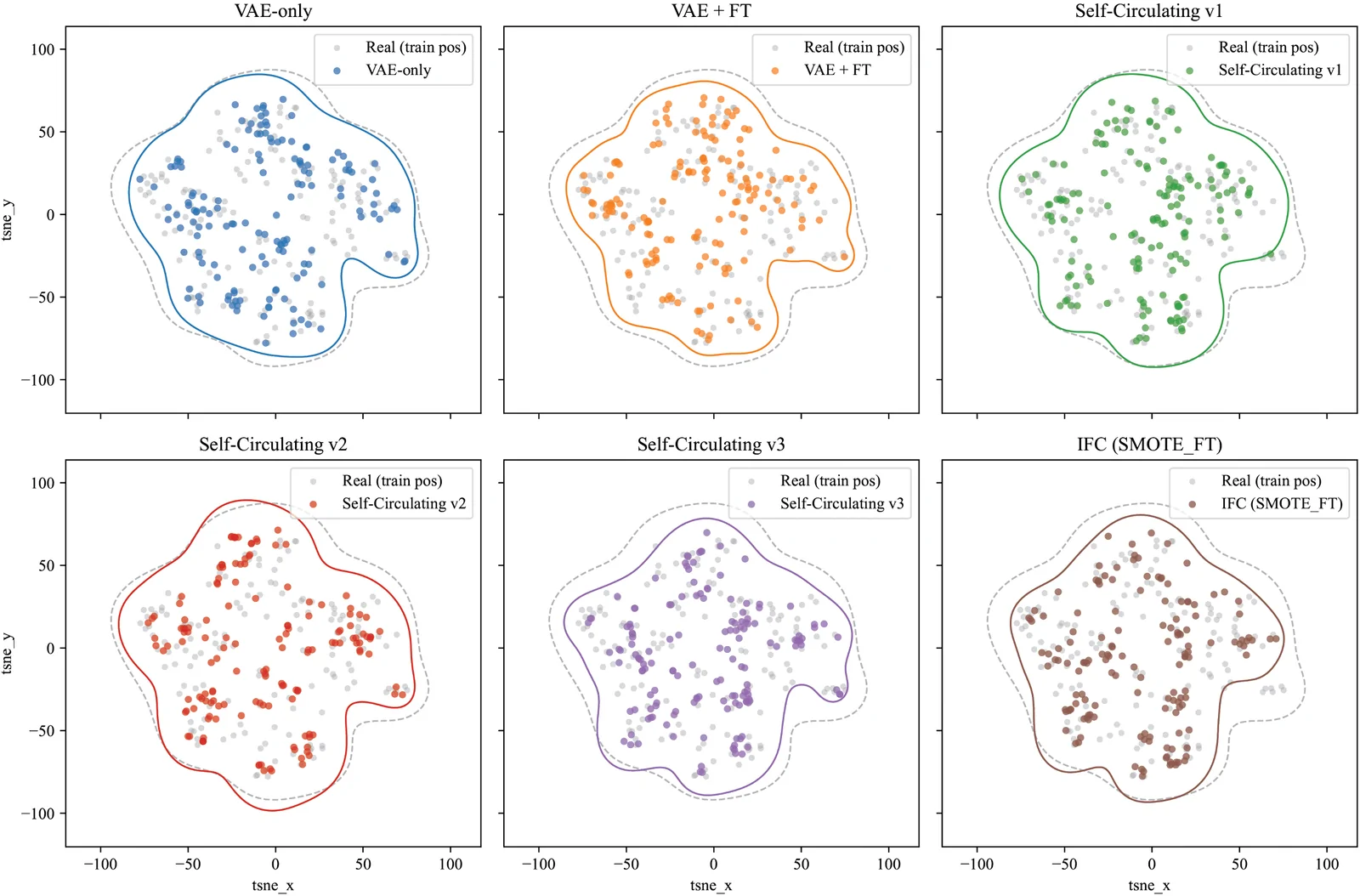
t-SNE visualization of IFC manifold evolution on FRM.

**Fig 9:**
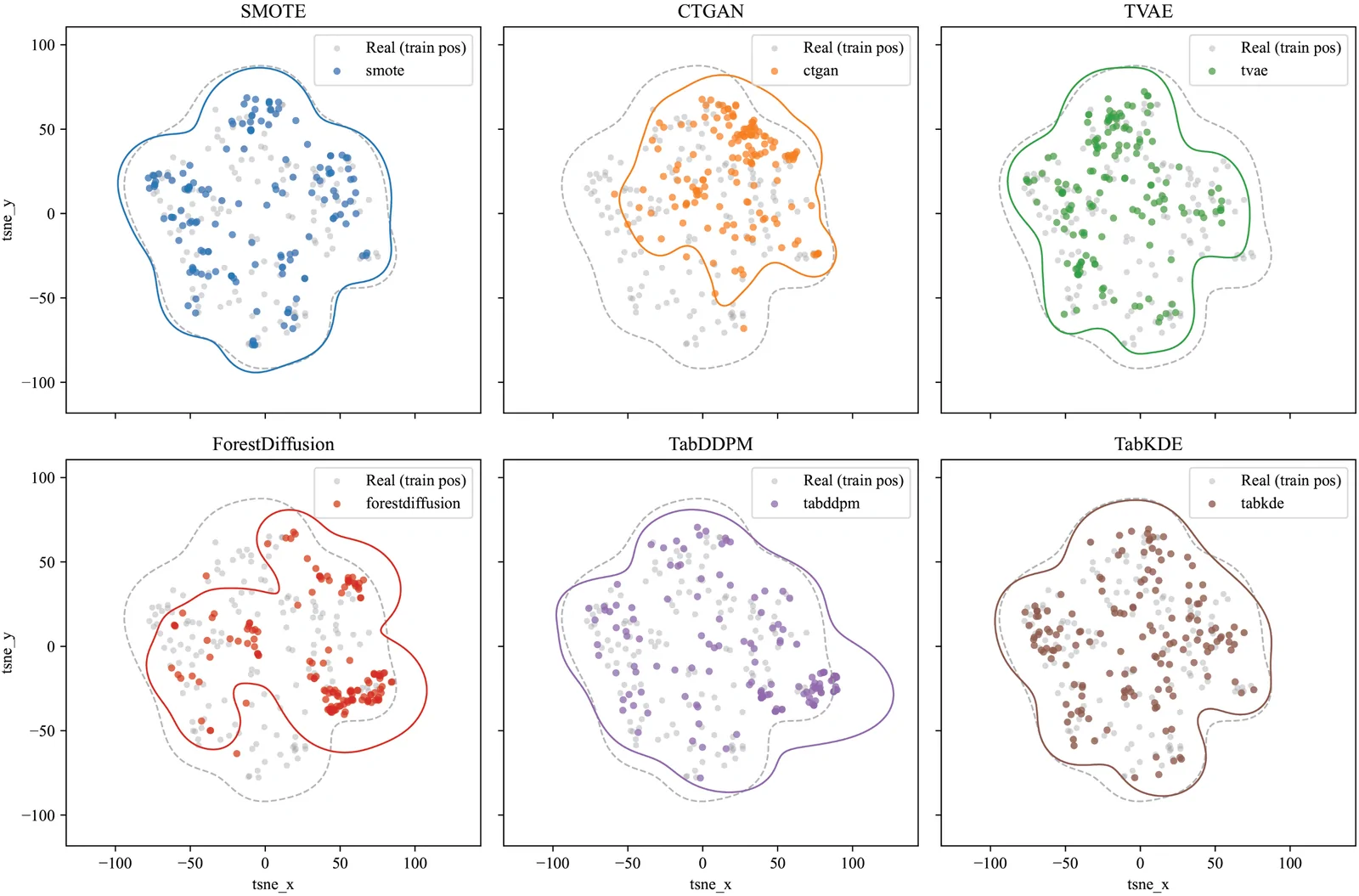
t-SNE comparison between IFC and baseline methods on FRM.

**Fig 10:**
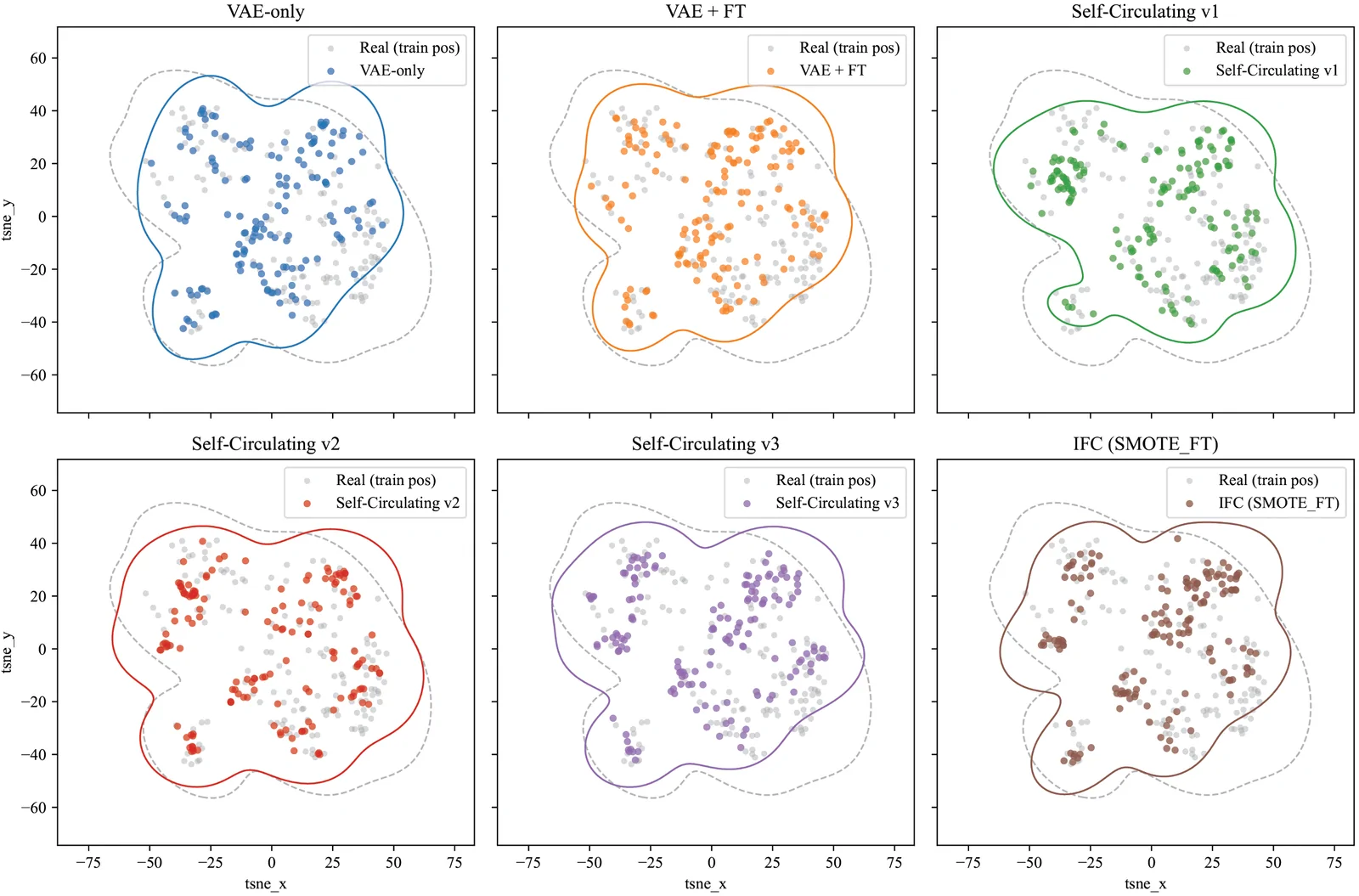
t-SNE visualization of IFC manifold evolution on PIMA.

**Fig 11:**
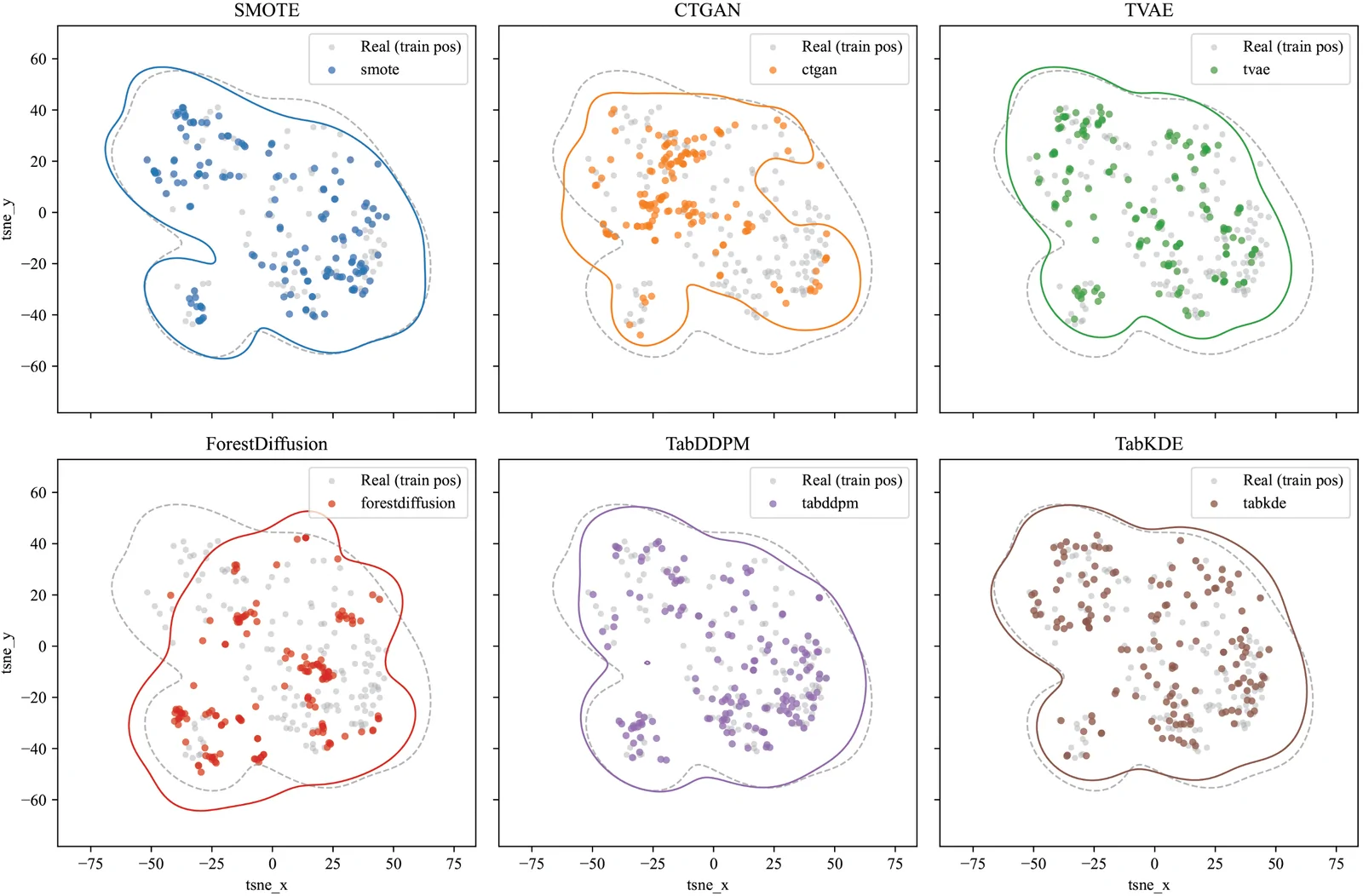
t-SNE comparison between IFC and baseline methods on PIMA.

**Manifold consistency.** Figs 8–11 visualize the evolution and comparison of synthetic minority-class manifolds on FRM and PIMA using t-SNE. All samples are projected jointly, with real samples shown in gray and synthetic samples overlaid in the same space. KDE contours are used to characterize the distribution boundary.

The manifold evolution of IFC shows progressive alignment toward the real minority-class distribution, with the self-circulating v2 stage providing the closest overlap. Among baseline methods, TabKDE and SMOTE generally preserve the manifold structure better, whereas CTGAN and TabDDPM frequently generate samples beyond or inside the real distribution boundary. ForestDiff performs slightly better on PIMA but remains among the weakest methods overall, particularly on FRM.

## Conclusion

This study proposed IFC-HFlowVAE, an iterative feedback framework for minority-class augmentation in heterogeneous medical tabular datasets. By integrating heterogeneous variational representation learning, flow-based latent refinement, and iterative sample updating, the proposed framework aims to generate synthetic samples that preserve minority-class characteristics while improving downstream predictive utility.

Extensive experiments on seven clinical datasets demonstrate that IFC-HFlowVAE achieves competitive classification performance compared with conventional oversampling methods and deep generative baselines. Fidelity analyses further show that the proposed framework can better preserve challenging minority-class characteristics, particularly complex numerical distributions that are difficult to reproduce with existing approaches.

Despite these promising results, further validation on larger and more diverse clinical datasets is required to assess the generalizability of IFC-HFlowVAE in real-world scenarios. Future work will investigate class-aware augmentation strategies that incorporate complementary information from majority classes and extend the framework toward more complex clinical data modalities.

## Supporting information

S1 Dataset PIMA Indians Diabetes dataset (CSV). Original data curated by the National Institute of Diabetes and Digestive and Kidney Diseases, released under a CC0: Public Domain license. Provided here as Supporting Information in case of the current community-maintained mirror being unstable.
(CSV)
