# Supplementary material for: IFC-HFlowVAE: A self-enhancing generative framework with structural anchoring for imbalanced clinical data augmentation
Source: PLoS One. 2026 Sep 3;21(9):e0357260. doi: 10.1371/journal.pone.0357260 (PMC13541264; doi:10.1371/journal.pone.0357260)
Supplement: Supplementary file 1 [file pone.0357260.s002.pdf]

# Response to Reviewers

**Manuscript ID: PONE-D-26-11647**

Dear Editor and Reviewers,

We sincerely thank the Editor and the reviewers for their careful evaluation and constructive suggestions. We greatly appreciate the reviewers' comments, which have helped us improve the clarity, rigor, and reproducibility of our manuscript.

In the revised manuscript, we have substantially improved the methodological description, experimental validation, and interpretation of the results. The major revisions include:

1. We redesigned the IFC framework description and added a clearer workflow illustration (Fig. 1) and a detailed HFlowVAE architecture illustration (Fig. 2). We also added an Implementation Details subsection to explicitly describe training procedures, model configurations, and experimental settings.
2. We expanded the experimental evaluation by adding repeated stratified five-fold cross-validation, ablation and sensitivity analyses, and more comprehensive fidelity analyses, including distributional fidelity, feature dependency evaluation, and manifold visualization.
3. We revised the discussion throughout the manuscript to avoid overinterpretation of conceptual mechanisms and better align the claims with experimentally supported evidence.
4. We added a new Data and Code Availability section. All seven datasets used in this study are now explicitly described, and the source code has been publicly released under the MIT License: https://github.com/JessieLu007/IFC-HFlowVAE
5. We revised the Conclusion section to include limitations and future research directions.

We hope that these revisions have addressed the reviewers' concerns and significantly improved the quality of the manuscript.

# Reviewer #1

## Comment 1:

The iterative feedback, structural anchors, and local GMM refinement mechanisms are theoretically interesting but difficult for readers to follow. Providing pseudocode or detailed flow diagrams, as well as explicit parameter selection criteria, would improve clarity and reproducibility.

## Response:

We appreciate this valuable suggestion. We agree that the original presentation of the iterative framework was not sufficiently clear for readers to fully understand the training procedure and implementation details.

In the revised manuscript, we redesigned the IFC workflow illustration (Fig. 1) and added a detailed HFlowVAE architecture diagram (Fig. 2) to provide a clearer overview of the proposed framework. We also added an Implementation Details subsection describing the training process, hyperparameter settings, and model configurations used in our experiments.

These revisions improve the reproducibility and accessibility of the proposed framework.

## Comment 2:

Current experiments only compare against CTGAN, TVAE, TabDDPM, and ForestDiffusion. Additional comparisons to methods such as GSN-HVNET and RGSB-UNet would strengthen the claims.

## Response:

We appreciate the reviewer for pointing out these representative works in medical AI. We have incorporated these studies into the revised Introduction to better position our work within the broader context of medical representation learning.

However, direct quantitative comparison with these approaches is not feasible because they are designed for medical image segmentation or classification tasks, whereas IFC-HFlowVAE focuses on minority-class synthetic data generation for heterogeneous clinical tabular datasets. The differences in data modality, learning objective, and evaluation protocol make a direct benchmark comparison inappropriate.

Therefore, we discuss these methods as related representation learning approaches rather than including them as direct baselines. For tabular clinical data augmentation, we expanded our comparison with representative generative baselines, including SMOTE, CTGAN, TVAE, ForestDiffusion, TabDDPM, and TabKDE.

## Comment 3:

There is insufficient discussion of dataset and task adaptability, particularly for high-dimensional mixed-type datasets.

## Response:

We appreciate this suggestion. In the revised manuscript, we expanded the discussion of dataset-specific performance differences, especially for datasets with heterogeneous numerical and categorical attributes. We further analyzed how feature characteristics influence generation quality and downstream classification performance.

## Comment 4:

Confidence intervals or statistical significance tests are not provided.

## Response:

We agree that additional statistical analysis can strengthen the reliability of minority-class performance evaluation. In the revised manuscript, we performed five repeated stratified five-fold cross-validation experiments and reported the mean and standard deviation of evaluation metrics. We also added statistical analyses to further assess performance differences between methods.

## Comment 5:

The t-SNE visualizations lack direct comparison with original samples and other models.

## Response:

We appreciate this comment. The original visualization already included real and synthetic samples projected into the same embedding space; however, this description was not sufficiently clear.

In the revised manuscript, we clarified the visualization procedure and added additional manifold comparisons. The new figures jointly visualize real samples and synthetic samples using the same dimensionality reduction space, allowing direct assessment of distribution alignment. We also included comparisons among IFC stages and baseline methods.

## Comment 6:

Updated references and deeper discussion of results are needed.

## Response:

We appreciate this suggestion. We have added and discussed the recommended related works, including RGGC-UNet, GSN-HVNET, and RGSB-UNet, in the revised Introduction.

Additionally, we substantially expanded the Results and Discussion section. Dataset-specific analyses were added to explain performance differences, and additional fidelity, dependency, and manifold analyses were introduced to provide deeper insight into the behavior of IFC-HFlowVAE.

# Reviewer #2

## Comment 1:

The manuscript is over-claimed. Terms such as "structural anchoring", "manifold stiffening", "discriminative sharpening", and "Model Autophagy Disorder" are not sufficiently supported by evidence.

## Response:

We sincerely appreciate this important comment. We agree that some conceptual descriptions in the original manuscript were stronger than what could be directly demonstrated experimentally.

Following this suggestion, we revised the manuscript to reduce speculative terminology and focus on experimentally supported descriptions. Several strong conceptual expressions have been removed or rewritten, and the discussion now emphasizes iterative refinement, minority-class representation preservation, and empirical validation rather than theoretical interpretation beyond the available evidence.

## Comment 2:

The experimental design requires stronger validation, including repeated splits, external validation, leakage checks, and sensitivity analysis.

## Response:

We appreciate this constructive suggestion. In the revised manuscript, we strengthened the experimental design in several aspects.

First, we performed repeated stratified five-fold cross-validation to evaluate the stability of the proposed framework. Second, we clarified the data processing pipeline and ensured that synthetic samples are generated only from the training folds, while validation and test folds remain completely isolated.

We would also like to clarify that no data leakage was present in the original experimental pipeline. Nevertheless, we have improved the description of the experimental procedure to make this separation explicit.

Due to the limited availability of suitable public clinical datasets with compatible formats, external validation was not performed in this study. We have added this point as a limitation and discussed it as an important direction for future research.

## Comment 3:

The theoretical discussion is weak, and the post-hoc XGBoost filtering step may bias synthetic samples toward the classifier.

## Response:

We appreciate this insightful observation. We have revised the theoretical discussion to avoid overstating assumptions and better align the theoretical motivation with the actual algorithmic contribution.

Furthermore, following the reviewer's suggestion, we completely removed the post-generation XGBoost filtering procedure from the revised framework. This modification eliminates the potential concern that generated samples may be optimized toward a specific downstream classifier rather than preserving intrinsic minority-class characteristics.

## Comment 4:

The authors may consider related works including the suggested references.

## Response:

We thank the reviewer for providing these references. We have incorporated and discussed these studies in the revised manuscript.
The suggested PLOS ONE study provides relevant background regarding the limitations of relying solely on conventional machine learning models for imbalanced medical data classification. The suggested IEEE Access study is related to VAE-based medical prediction approaches and has been discussed in the context of VAE-based learning strategies.
These additions help better position our work within existing medical AI research.

## Final Response

We sincerely thank the Editor and both reviewers again for their valuable comments. The suggestions have greatly helped us improve the manuscript by strengthening methodological clarity, experimental rigor, and the interpretation of our findings. We believe that the revised manuscript provides a clearer and more comprehensive presentation of IFC-HFlowVAE and its potential for minority-class augmentation in heterogeneous clinical tabular datasets.
