# Supplementary material for: IFC-HFlowVAE: A self-enhancing generative framework with structural anchoring for imbalanced clinical data augmentation
Source: PLoS One. 2026 Sep 3;21(9):e0357260. doi: 10.1371/journal.pone.0357260 (PMC13541264; doi:10.1371/journal.pone.0357260)
Supplement: Peer review history

# Peer review: Decision Letter 0

**DOI:** 10.1371/journal.pone.0357260.r001

**Authors:** Zeheng Wang

15 Jun 2026

Dear Dr. Chen,

Thank you for submitting your manuscript to PLOS ONE. After careful consideration, we feel that it has merit but does not fully meet PLOS ONE’s publication criteria as it currently stands. Therefore, we invite you to submit a revised version of the manuscript that addresses the points raised during the review process.

Please submit your revised manuscript by Jul 30 2026 11:59PM. If you will need more time than this to complete your revisions, please reply to this message or contact the journal office at plosone@plos.org. When you're ready to submit your revision, log on to https://www.editorialmanager.com/pone/ and select the 'Submissions Needing Revision' folder to locate your manuscript file.

- A letter that responds to each point raised by the academic editor and reviewer(s). You should upload this letter as a separate file labeled 'Response to Reviewers'.
- A marked-up copy of your manuscript that highlights changes made to the original version. You should upload this as a separate file labeled 'Revised Manuscript with Track Changes'.
- An unmarked version of your revised paper without tracked changes. You should upload this as a separate file labeled 'Manuscript'.

If you would like to make changes to your financial disclosure, please include your updated statement in your cover letter. Guidelines for resubmitting your figure files are available below the reviewer comments at the end of this letter.

If applicable, we recommend that you deposit your laboratory protocols in protocols.io to enhance the reproducibility of your results. Protocols.io assigns your protocol its own identifier (DOI) so that it can be cited independently in the future. For instructions see: https://journals.plos.org/plosone/s/submission-guidelines#loc-laboratory-protocols. Additionally, PLOS ONE offers an option for publishing peer-reviewed Lab Protocol articles, which describe protocols hosted on protocols.io. Read more information on sharing protocols at https://plos.org/protocols?utm_medium=editorial-email&utm_source=authorletters&utm_campaign=protocols.

As the corresponding author, your ORCID iD is verified in the submission system and will appear in the published article. PLOS supports the use of ORCID, and we encourage all coauthors to register for an ORCID iD and use it as well. Please encourage your coauthors to verify their ORCID iD within the submission system before final acceptance, as unverified ORCID iDs will not appear in the published article. *Only*  the individual author can complete the verification step; PLOS staff *cannot*  verify ORCID iDs on behalf of authors.

We look forward to receiving your revised manuscript.

Kind regards,

Zeheng Wang

Academic Editor

PLOS One

Journal requirements:

When submitting your revision, we need you to address these additional requirements.

1. Please ensure that your manuscript meets PLOS ONE's style requirements, including those for file naming. The PLOS ONE style templates can be found at

https://journals.plos.org/plosone/s/file?id=wjVg/PLOSOne_formatting_sample_main_body.pdf and

https://journals.plos.org/plosone/s/file?id=ba62/PLOSOne_formatting_sample_title_authors_affiliations.pdf

2. Please note that PLOS One has specific guidelines on code sharing for submissions in which author-generated code underpins the findings in the manuscript. In these cases, we expect all author-generated code to be made available without restrictions upon publication of the work. Please review our guidelines at https://journals.plos.org/plosone/s/materials-and-software-sharing#loc-sharing-code and ensure that your code is shared in a way that follows best practice and facilitates reproducibility and reuse.

If the reviewer comments include a recommendation to cite specific previously published works, please review and evaluate these publications to determine whether they are relevant and should be cited. There is no requirement to cite these works unless the editor has indicated otherwise.

Additional Editor Comments:

Please cite only those references necessary to the study if the reviewers request.

[Note: HTML markup is below. Please do not edit.]

Reviewers' comments:

Reviewer's Responses to Questions

**Comments to the Author**

1. Is the manuscript technically sound, and do the data support the conclusions?

Reviewer #1: Yes

Reviewer #2: Partly

**********

2. Has the statistical analysis been performed appropriately and rigorously?

Reviewer #1: Yes

Reviewer #2: No

**********

3. Have the authors made all data underlying the findings in their manuscript fully available?

The PLOS Data policy

Reviewer #1: Yes

Reviewer #2: No

**********

4. Is the manuscript presented in an intelligible fashion and written in standard English?

Reviewer #1: Yes

Reviewer #2: Yes

**********

Reviewer #1: The manuscript presents a novel framework, IFC-HFlowVAE, for imbalanced clinical data augmentation. The study is well-motivated, and the proposed approach, integrating iterative feedback, structural anchors, and GMM-based local refinement, shows promise in improving minority-class recognition across multiple datasets. The experimental results indicate clear gains in F1-score and G-mean over baseline generative models. However, the manuscript has several weaknesses that limit its clarity, reproducibility, and the strength of its claims. Key concerns include insufficient comparison to relevant recent methods, overly complex methodological descriptions, limited visualizations, and incomplete discussion of results. These issues must be addressed before the work can be considered for publication.

1. Complex methodology description and reproducibility concerns

The iterative feedback, structural anchors, and local GMM refinement mechanisms are theoretically interesting but difficult for readers to follow. Providing pseudocode or detailed flow diagrams, as well as explicit parameter selection criteria (e.g., SMOTE seed weights, number of iterations, GMM clusters), would improve clarity and reproducibility and allow fair comparison with baseline models such as RGGC-UNet.

2. Limited baseline comparisons

Current experiments only compare against CTGAN, TVAE, TabDDPM, and ForestDiffusion. Given the focus on clinical data augmentation and minority-class learning, additional comparisons to state-of-the-art segmentation or augmentation frameworks such as GSN-HVNET and RGSB-UNet would strengthen claims about generalization across numerical, categorical, and image-based datasets.

Insufficient discussion on dataset and task adaptability

While performance is reported for BCC, PIMA, and THY datasets, there is little explanation for the performance differences in high-dimensional mixed-type datasets (e.g., FRM). The authors should analyze the impact of different feature types (numerical vs categorical vs image) on their method and compare to RGGC-UNet’s multi-scale feature handling capabilities.

3. Evaluation metrics and interpretability

F1-score and G-mean are used as evaluation metrics, but confidence intervals or statistical significance tests are not provided. Including these analyses would strengthen the reliability of the results, particularly for minority-class performance, and allow comparison with RGSB-UNet’s decision boundary interpretability.

4. Insufficient visualization

t-SNE visualizations illustrate iterative convergence but lack direct comparison with original samples or other models (e.g., GSN-HVNET), making it difficult to visually assess the “discriminative sharpening” effect. Additional visualizations should be provided to show how synthetic samples improve minority-class representation.

5. Updated references needed

Most references focus on generative models such as CTGAN and TabDDPM. The authors should cite and discuss RGGC-UNet, GSN-HVNET, and RGSB-UNet, explaining how IFC-HFlowVAE differs or complements these recent approaches in medical image processing.

6. Deeper discussion of results required

For datasets like DRP and FRM, the manuscript highlights substantial G-mean improvements but does not analyze why. The authors should provide quantitative discussion of minority-class feature distributions, iterative feedback effects, and local GMM refinement, and compare with RGSB-UNet’s strategies for few-sample enhancement.

Reviewer #2: The manuscript is currently over-claimed and not sufficiently rigorous for a clinical data augmentation paper. Many terms, such as “structural anchoring,” “manifold stiffening,” “discriminative sharpening,” and “Model Autophagy Disorder,” are used in a very heavy way, but the paper does not clearly prove that these mechanisms are actually happening. Much of the argument reads more like conceptual storytelling than demonstrated evidence. The authors should either provide direct quantitative evidence for these claimed mechanisms or reduce the strength of these claims.

The experimental design is also not convincing enough. The datasets are small public benchmark datasets, but the manuscript presents the results as if the method has broad clinical reliability. The paper needs stronger validation, including repeated stratified splits, external validation if possible, leakage checks, sensitivity analysis for key hyperparameters, and clearer reporting of how synthetic samples are generated only from the training fold. Without this, the reported gains may simply reflect overfitting, classifier-specific artifacts, or favorable data splitting rather than a genuinely better augmentation method.

The theoretical section is weak and does not really support the proposed method. The KL decomposition and Rademacher-complexity discussion are presented in a rather loose way, and several assumptions are not justified for real clinical tabular data. The post-hoc XGBoost filtering step is also problematic because it may bias the synthetic data toward the downstream classifier rather than producing clinically meaningful samples. Overall, the authors need to tone down the theory, clarify the actual algorithmic contribution, and provide stronger ablation studies to show which component is truly necessary.

The authors may be interested in these related works for reference: 10.1371/journal.pone.0298328 and 10.1109/ACCESS.2023.3348810.

**********

what does this mean?). If published, this will include your full peer review and any attached files.

If you choose “no”, your identity will remain anonymous but your review may still be made public.

**Do you want your identity to be public for this peer review?**  For information about this choice, including consent withdrawal, please see our Privacy Policy

Reviewer #1: No

Reviewer #2: No

**********

[NOTE: If reviewer comments were submitted as an attachment file, they will be attached to this email and accessible via the submission site. Please log into your account, locate the manuscript record, and check for the action link "View Attachments". If this link does not appear, there are no attachment files.]

To ensure your figures meet our technical requirements, please review our figure guidelines: https://journals.plos.org/plosone/s/figures

You may also use PLOS’s free figure tool, NAAS, to help you prepare publication quality figures: https://journals.plos.org/plosone/s/figures#loc-tools-for-figure-preparation.

NAAS will assess whether your figures meet our technical requirements by comparing each figure against our figure specifications.

# Author comment: Author response to Decision Letter 1

**DOI:** 10.1371/journal.pone.0357260.r002

5 Aug 2026

Reviewer #1

Comment 1:

The iterative feedback, structural anchors, and local GMM refinement mechanisms are theoretically interesting but difficult for readers to follow. Providing pseudocode or detailed flow diagrams, as well as explicit parameter selection criteria, would improve clarity and reproducibility.

Response:

We appreciate this valuable suggestion. We agree that the original presentation of the iterative framework was not sufficiently clear for readers to fully understand the training procedure and implementation details.

In the revised manuscript, we redesigned the IFC workflow illustration (Fig. 1) and added a detailed HFlowVAE architecture diagram (Fig. 2) to provide a clearer overview of the proposed framework. We also added an Implementation Details subsection describing the training process, hyperparameter settings, and model configurations used in our experiments.

These revisions improve the reproducibility and accessibility of the proposed framework.

Comment 2:

Current experiments only compare against CTGAN, TVAE, TabDDPM, and ForestDiffusion. Additional comparisons to methods such as GSN-HVNET and RGSB-UNet would strengthen the claims.

Response:

We appreciate the reviewer for pointing out these representative works in medical AI. We have incorporated these studies into the revised Introduction to better position our work within the broader context of medical representation learning.

However, direct quantitative comparison with these approaches is not feasible because they are designed for medical image segmentation or classification tasks, whereas IFC-HFlowVAE focuses on minority-class synthetic data generation for heterogeneous clinical tabular datasets. The differences in data modality, learning objective, and evaluation protocol make a direct benchmark comparison inappropriate.

Therefore, we discuss these methods as related representation learning approaches rather than including them as direct baselines. For tabular clinical data augmentation, we expanded our comparison with representative generative baselines, including SMOTE, CTGAN, TVAE, ForestDiffusion, TabDDPM, and TabKDE.

Comment 3:

There is insufficient discussion of dataset and task adaptability, particularly for high-dimensional mixed-type datasets.

Response:

We appreciate this suggestion. In the revised manuscript, we expanded the discussion of dataset-specific performance differences, especially for datasets with heterogeneous numerical and categorical attributes. We further analyzed how feature characteristics influence generation quality and downstream classification performance.

Comment 4:

Confidence intervals or statistical significance tests are not provided.

Response:

We agree that additional statistical analysis can strengthen the reliability of minority-class performance evaluation. In the revised manuscript, we performed five repeated stratified five-fold cross-validation experiments and reported the mean and standard deviation of evaluation metrics. We also added statistical analyses to further assess performance differences between methods.

Comment 5:

The t-SNE visualizations lack direct comparison with original samples and other models.

Response:

We appreciate this comment. The original visualization already included real and synthetic samples projected into the same embedding space; however, this description was not sufficiently clear.

In the revised manuscript, we clarified the visualization procedure and added additional manifold comparisons. The new figures jointly visualize real samples and synthetic samples using the same dimensionality reduction space, allowing direct assessment of distribution alignment. We also included comparisons among IFC stages and baseline methods.

Comment 6:

Updated references and deeper discussion of results are needed.

Response:

We appreciate this suggestion. We have added and discussed the recommended related works, including RGGC-UNet, GSN-HVNET, and RGSB-UNet, in the revised Introduction.

Additionally, we substantially expanded the Results and Discussion section. Dataset-specific analyses were added to explain performance differences, and additional fidelity, dependency, and manifold analyses were introduced to provide deeper insight into the behavior of IFC-HFlowVAE.

Reviewer #2

Comment 1:

The manuscript is over-claimed. Terms such as “structural anchoring”, “manifold stiffening”, “discriminative sharpening”, and “Model Autophagy Disorder” are not sufficiently supported by evidence.

Response:

We sincerely appreciate this important comment. We agree that some conceptual descriptions in the original manuscript were stronger than what could be directly demonstrated experimentally.

Following this suggestion, we revised the manuscript to reduce speculative terminology and focus on experimentally supported descriptions. Several strong conceptual expressions have been removed or rewritten, and the discussion now emphasizes iterative refinement, minority-class representation preservation, and empirical validation rather than theoretical interpretation beyond the available evidence.

Comment 2:

The experimental design requires stronger validation, including repeated splits, external validation, leakage checks, and sensitivity analysis.

Response:

We appreciate this constructive suggestion. In the revised manuscript, we strengthened the experimental design in several aspects.

First, we performed repeated stratified five-fold cross-validation to evaluate the stability of the proposed framework. Second, we clarified the data processing pipeline and ensured that synthetic samples are generated only from the training folds, while validation and test folds remain completely isolated.

We would also like to clarify that no data leakage was present in the original experimental pipeline. Nevertheless, we have improved the description of the experimental procedure to make this separation explicit.

Due to the limited availability of suitable public clinical datasets with compatible formats, external validation was not performed in this study. We have added this point as a limitation and discussed it as an important direction for future research.

Comment 3:

The theoretical discussion is weak, and the post-hoc XGBoost filtering step may bias synthetic samples toward the classifier.

Response:

We appreciate this insightful observation. We have revised the theoretical discussion to avoid overstating assumptions and better align the theoretical motivation with the actual algorithmic contribution.

Furthermore, following the reviewer’s suggestion, we completely removed the post-generation XGBoost filtering procedure from the revised framework. This modification eliminates the potential concern that generated samples may be optimized toward a specific downstream classifier rather than preserving intrinsic minority-class characteristics.

Comment 4:

The authors may consider related works including the suggested references.

Response:

We thank the reviewer for providing these references. We have incorporated and discussed these studies in the revised manuscript.

The suggested PLOS ONE study provides relevant background regarding the limitations of relying solely on conventional machine learning models for imbalanced medical data classification. The suggested IEEE Access study is related to VAE-based medical prediction approaches and has been discussed in the context of VAE-based learning strategies.

These additions help better position our work within existing medical AI research.

Final Response

We sincerely thank the Editor and both reviewers again for their valuable comments. The suggestions have greatly helped us improve the manuscript by strengthening methodological clarity, experimental rigor, and the interpretation of our findings. We believe that the revised manuscript provides a clearer and more comprehensive presentation of IFC-HFlowVAE and its potential for minority-class augmentation in heterogeneous clinical tabular datasets.

Attachment Submitted filename: Response to Reviewers.pdf

# Peer review: Decision Letter 1

**DOI:** 10.1371/journal.pone.0357260.r003

**Authors:** Zeheng Wang

16 Aug 2026

IFC-HFlowVAE: A self-enhancing generative framework with structural anchoring for imbalanced clinical data augmentation

PONE-D-26-11647R1

Dear Dr. Chen,

We’re pleased to inform you that your manuscript has been judged scientifically suitable for publication and will be formally accepted for publication once it meets all outstanding technical requirements.

Within one week, you’ll receive an e-mail detailing the required amendments. When these have been addressed, you’ll receive a formal acceptance letter and your manuscript will be scheduled for publication.

An invoice will be generated when your article is formally accepted. Please note, if your institution has a publishing partnership with PLOS and your article meets the relevant criteria, all or part of your publication costs will be covered. Please make sure your user information is up-to-date by logging into Editorial Manager at Editorial Manager® and clicking the ‘Update My Information' link at the top of the page. For questions related to billing, please contact billing support.

If your institution or institutions have a press office, please notify them about your upcoming paper to help maximize its impact. If they’ll be preparing press materials, please inform our press team as soon as possible -- no later than 48 hours after receiving the formal acceptance. Your manuscript will remain under strict press embargo until 2 pm Eastern Time on the date of publication. For more information, please contact onepress@plos.org.

Kind regards,

Zeheng Wang

Academic Editor

PLOS One

Additional Editor Comments (optional):

Reviewers' comments:

Reviewer's Responses to Questions

**Comments to the Author**

Reviewer #1: All comments have been addressed

**********

2. Is the manuscript technically sound, and do the data support the conclusions?

Reviewer #1: Yes

**********

3. Has the statistical analysis been performed appropriately and rigorously?

Reviewer #1: Yes

**********

4. Have the authors made all data underlying the findings in their manuscript fully available?

The PLOS Data policy

Reviewer #1: Yes

**********

5. Is the manuscript presented in an intelligible fashion and written in standard English?

Reviewer #1: Yes

**********

Reviewer #1: The authors have addressed all my comments, and the manuscript has been revised accordingly. I believe that the manuscript is now suitable for publication and recommend its acceptance.

**********

what does this mean?). If published, this will include your full peer review and any attached files.

If you choose “no”, your identity will remain anonymous but your review may still be made public.

**Do you want your identity to be public for this peer review?**  For information about this choice, including consent withdrawal, please see our Privacy Policy

Reviewer #1: No

**********

# Editor report: Acceptance letter

**DOI:** 10.1371/journal.pone.0357260.r004

**Authors:** Zeheng Wang

PONE-D-26-11647R1

PLOS One

Dear Dr. Chen,

I'm pleased to inform you that your manuscript has been deemed suitable for publication in PLOS One. Congratulations! Your manuscript is now being handed over to our production team.

At this stage, our production department will prepare your paper for publication. This includes ensuring the following:

* All references, tables, and figures are properly cited

* All relevant supporting information is included in the manuscript submission,

* There are no issues that prevent the paper from being properly typeset

You will receive further instructions from the production team, including instructions on how to review your proof when it is ready. Please keep in mind that we are working through a large volume of accepted articles, so please give us a few days to review your paper and let you know the next and final steps.

Lastly, if your institution or institutions have a press office, please let them know about your upcoming paper now to help maximize its impact. If they'll be preparing press materials, please inform our press team within the next 48 hours. Your manuscript will remain under strict press embargo until 2 pm Eastern Time on the date of publication. For more information, please contact onepress@plos.org.

You will receive an invoice from PLOS for your publication fee after your manuscript has reached the completed accept phase. If you receive an email requesting payment before acceptance or for any other service, this may be a phishing scheme. Learn how to identify phishing emails and protect your accounts at https://explore.plos.org/phishing.

If we can help with anything else, please email us at customercare@plos.org.

Thank you for submitting your work to PLOS One and supporting open access.

Kind regards,

PLOS One Editorial Office Staff

on behalf of

Dr. Zeheng Wang

Academic Editor

PLOS One
